# Two‐photon Excitation of Bright Diaza[4]Helicenes for Isotropic and Circularly Polarized Emission

**DOI:** 10.1002/chem.202501212

**Published:** 2025-05-02

**Authors:** Bibiana Fabri, Davide F. De Rosa, Dominic J. Black, Rebecca Mucci, Artemijs Krimovs, Robert Pal, Jérôme Lacour

**Affiliations:** ^1^ Department of Organic Chemistry University of Geneva Quai Ernest Ansermet 30 Geneva 4 1211 Switzerland; ^2^ Department of Chemistry Durham University Durham DH1 3LE UK

**Keywords:** bright fluorophores, chiroptical properties, circularly polarized luminescence, microscopy, two‐photon excitation

## Abstract

Helicenes are chiral organic dyes that are attracting growing attention due to the high tunability of their (chir)optical and electronic properties. In this work, a series of functionalized cationic diaza[4]helicenes based on dimethoxyquinacridinium (DMQA) scaffolds are presented. By merging branched N‐alkyl side chains and triple *para*‐functionalization with OMe groups, structures combine improved chiroptical responses (5x increase) and strong fluorescence quantum yields (Φ_f_ ≈ 70% in acetonitrile). An overall improved efficiency of the emission of circularly polarized light with *B*_CPL_ values reaching 3.4 M^−1^ cm^−1^ is obtained. Additionally, two‐photon excitation (2PE) studies were performed, showing good values of cross section (CS) at 810 nm. Interestingly, 2PE circularly polarized luminescence (CPL) spectra were acquired for the most performant derivatives (N‐isopropyl and N‐cyclohexyl); this type of measurement being usually challenging for organic molecular species. Finally, the viability of these compounds in single‐photon (1PE) and 2PE microscopy is also shown.

## Graphical abstract

Tris‐*p*‐OMe cationic diaza[4]helicenes with improved (CPL) brightness (up to 3.4 M^−1^ cm^−1^ for **2d**) are achieved by combining high Φ_f_ and enhanced chiroptical properties arising with branched N‐substituents. Efficient 2PE at 810 nm and 2PE CPL spectra are reported, the latter being uncommon for small organic molecules. Their viability in cell imaging by 1PE and 2PE confocal microscopy was also proven.

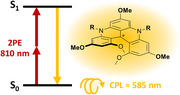

## 1 Introduction

**Figure 1:**
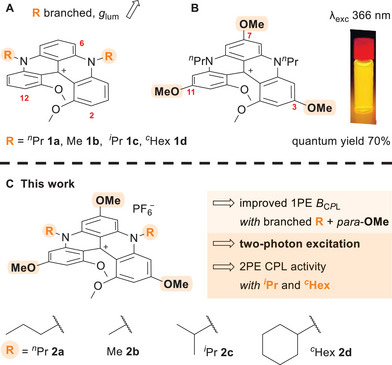
A) cationic diaza[4]helicenes **1** with various N‐alkyl chains; B) previously reported triple *para*‐OMe derivative **2a**; C) *para*‐OMe derivatives with linear (**2a**–**2b**) and branched (**2c**–**2d**) alkyl chains.

Chiral organic dyes represent attractive research targets for the control of chiroptoelectronic properties, such as electronic circular dichroism (ECD) and circularly polarized luminescence (CPL), which in turn can be useful in various applications (e.g., photonics, optoelectronic devices).^[^ ^1^ ^]^ Among chiral dyes, helicenes generated an increasing interest due to the diversity of possible chemical structures and their strong interaction with polarized light.^[^ ^2^ ^]^ In this context, cationic diaza[4]helicenes of dimethoxyquinacridinium (DMQA) type (**1**, (Figure 1A) are a particularly versatile class of derivatives.

They are readily prepared and resolved into single enantiomers of (*M*) and (*P*) configurations, which present high configurational stability (Δ*G* ^‡^_racem_ ∼ 42 kcal mol^−1^).^[^ ^3^ ^]^ Furthermore, these structures are suited for regioselective late‐stage functionalization strategies introducing substituents either in *meta* to the formal positive charge (positions 6 and 2/12), or more recently, in *para* (positions 3, 7, and 11).^[^ ^4^ ^]^ Diverse functional groups can then be placed on the outer rim of the helical core allowing an extensive tuning of key photophysical and electronic properties of the dyes. Nowadays, these small [4]helicenes have found applications as catalysts, electrolytes in organic redox flow batteries, spin filters, and bioprobes.^[^ ^5^ ^]^ In addition, they have been proven good candidates for obtaining persistent neutral radicals.^[^ ^4^, ^6^ ^]^ Most commonly, helicenes based on scaffold **1** absorb and emit in the red domain of the visible spectrum (> 600 nm), with moderate to good quantum yields (5–50%).^[^ ^4a,c^ ^]^ Recently, it was reported that, by adding three *para*‐OMe groups on the helical scaffold (**2a**, (Figure 1B), a fluorescence quantum yield of 70% can be achieved, accompanied by a hypsochromic shift of the optical properties (**1a** *λ*_max_ = 616 nm, *λ*_em_ = 667 nm → **2a** *λ*_max_ = 533 nm, *λ*_em_ = 580 nm).^[^ ^4d^ ^]^

For imaging purposes, the possibility to excite compounds at low energy, and ideally in the Near Infrared (NIR) domain, is an attractive possibility. In this regard, one solution is to excite the compound using two simultaneously absorbed photons of half the energy (double wavelength) via a multiphoton (MP) process.^[^ ^7^ ^]^ Two‐photon excitation (2PE) enables, at the same time, good tissue penetration of biological media and high spatial resolution, which make it widely employed in optical imaging.^[^ ^8^ ^]^ In 2PE, the probability of two photons to be absorbed simultaneously is dependent on the light intensity and the cross section (CS, indicated by σ^2^) of the compound, with Goeppert‐Mayer (GM) units, where 1GM = 10^−50^ cm^4^ s photon^−1^.^[^ ^7^, ^9^ ^]^ Several organic derivatives able to undergo 2PE, even with very high values of CS (> 1000 GM), are known.^[^ ^10^ ^]^ Even though 2PE of helical derivatives has been already reported, complemented in some cases by measurements of two‐photon ECD spectra and one‐photon excitation (1PE) CPL activity,^[^ ^11^ ^]^ reports of 2PE CPL are seldom due to instrumental constrains and have only recently appeared including perovskites and liquid crystals.^[^ ^12^ ^]^ In the context of molecular emitters, the work of Pal and co‐workers on lanthanide complexes holds a pivotal role.^[^ ^13^ ^]^ Additionally, it was demonstrated the importance of CPL confocal microscopy in extracting additional information, thanks to the polarization of the luminescent probe, making 2PE CPL‐active probes particularly desirable.^[^ ^13a,c^ ^]^ However, aside from few lanthanide complexes, molecular, and especially organic, systems able to perform such task are not yet readily available.

Herein, *para*‐OMe diaza[4]helicenes **2a**–**2d** (Figure 1C) are presented bearing primary and secondary alkyl chains on the N‐atoms. While the introduction of methoxy (OMe) groups *para* to the formal positive charge of **1** affords fluorescent quantum yields (Φ_f_) around 70%,^[^ ^4d^ ^]^ higher chiroptical responses were looked‐for by including N‐isopropyl (*^i^* Pr, **2c**) and N‐cyclohexyl (*^c^* Hex, **2d**) chains, known to give higher *g* factors compared to the more classical N‐*^n^* Pr chains.^[^ ^3^, ^14^ ^]^ In fact, the steric interactions between the branched N‐alkyl chains and the helical skeleton provokes a local strain and a concomitant pyramidalization of the nitrogen bridging atoms. This distortion is then at the origin of the enhanced chiroptical properties, as supported by TD‐DFT calculations.^[^ ^14^ ^]^ Thanks to the combination of high Φ_f_ and increased *g*_lum_ (≥ 5 × 10^−4^), single‐photon (1PE) CPL brightness (*B*_CPL_)^[^ ^15^ ^]^ values of 2.6 and 3.4 M^−1^ cm^−1^ are reached with novel compounds **2c** and **2d**, respectively, which are among the highest obtained for this class of cationic [n]helicenes.^[^ ^15^ ^]^ Additionally, derivatives **2a**–**2d** are employed in 2PE studies, giving good values of CS at 810 nm (σ^2^ up to 137 GM). Again, branched N‐alkyl derivatives are best, with the highest values of CS. This, combined with favorable *g* factors, allows for an efficient measurement of CPL spectra via 2PE. Noteworthy 2PE *B*_CPL_ values of 0.03 and 0.05 GM are obtained with these small organic derivatives.^[^ ^13a^ ^]^ Finally, microscopy studies using **2d** (strongest CS) as probe are presented.

## 2 Results and Discussion

### 2.1 Synthesis

**Scheme 1:**
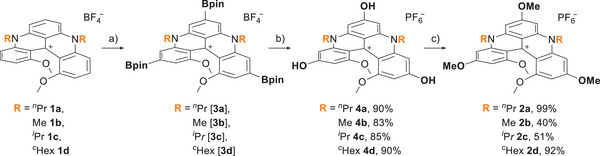
Synthesis of triple *para*‐OMe diaza[4]helicenes with differentiated nitrogen substituents. Conditions: a) [Ir(cod)OMe]_2_ (cod = 1,5‐cyclooctadiene), 3,4,7,8‐tetramethyl‐1,10‐phenanthroline, bis(pinacolato)diboron (B_2_pin_2_), dry THF, 80 °C, 16 hours; b) (1) H_2_O_2_ (aq), NaOH, THF, RT, 16 hours, (2) anion metathesis with KPF_6_ (aq); c) MeI, Cs_2_CO_3_, DMF, RT, 16 hours.

Racemic compounds **1a**–**1d** were synthesized following reported guidelines,^[^ ^14^, ^16^ ^]^ and used as substrates to obtain *para*‐OMe **2a**–**2d**, following synthetic steps previously developed for **2a** (Scheme 1).^[^ ^4d^ ^]^ Synthetically, pinacol boronic ester (Bpin) moieties were first introduced in *para* positions by Ir‐catalyzed direct C─H borylations (step a) resulting in intermediates of type **3**. Due to a lack of stability and fast protodeboronation of **3a–3d**, these were subjected directly to hydroxylation conditions (H_2_O_2_/NaOH) affording tris(OH) derivatives **4** in 83–90% yields (step b).^[^ ^17^ ^]^ Then, alkylation with methyl iodide (excess) under basic conditions was performed giving methylated analogues **2** (step c). While this step is effective for **2a** and **2d** (>90%, R = *^n^* Pr, *^c^* Hex), it is less successful for compounds **2b** (R = Me) and **2c** (R = *^i^* Pr), which were obtained in only 40 and 51% yields, respectively (Scheme 1). The difference in reactivity is most probably linked to the variations in solubility of the tris(OH) species **4** and their deprotonated forms. In fact, with Me or *^i^* Pr chains, the structures possess a lower lipophilicity than with longer residues, *^n^* Pr or *^c^* Hex, making derivatives **2b** and **2c** less soluble in the reaction mixture. Moreover, upon deprotonation, precipitation of **4b** and **4c** occurs. Overall, a short series of *para*‐functionalized OMe derivatives **2** was synthesized starting from the corresponding racemic **1**. While (*M*) and (*P*) enantiomers of **2a** were already available from a previous project,^[^ ^4d^ ^]^ enantiopure **2b**, **2c,** and **2d** were obtained by chiral stationary phase‐HPLC resolution of the racemates using a semi‐preparative CHIRALPAK IC column (see Supporting Information).

### 2.2 Chiroptical Properties

Absorption and emission of newly synthesized dyes **2b**–**2d** were studied in acetonitrile at room temperature and compared to **2a** (Figure S5). Overall, optical properties varied as expected from previous studies on unfunctionalized derivatives **1**.^[^ ^14^, ^18^ ^]^ The presence of N‐Me side chains, instead of N‐*^n^* Pr, had no major impact, with the absorption and emission spectra of **2b** being almost superimposable to those of **2a**. On the contrary, the strain induced by the N‐*^i^* Pr and N‐*^c^* Hex side chains brought about a slight red‐shift in both absorption and emission (Table 1). All four *para*‐OMe helicenes are equally efficient fluorophores, with Φ_f_ ranging from 70 to 75% and τ of 16 to 17 ns (Table 1), these differences being within the experimental error. Consequently, the rates of radiative (*k*_r_) and nonradiative (*k*_nr_) decay are similar for all four derivatives. The *para*‐electron donating group effect of the methoxy substituents, described previously for **2a**,^[^ ^4d^ ^]^ applies hence to **2b**, **2c,** and **2d**. Molecular engineering of compounds **1**→**2** is therefore quite predictive irrespective of the different side chains.^[^ ^14^, ^18^ ^]^

**Table 1:** (Chir)optical properties of *para*‐OMe diaza[4]helicenes 2 in acetonitrile.

Compound^[^ ^a^ ^]^	*λ_abs_* ^max^ (nm)	ε (M^−1^ cm^−1^)	*λ*_em_ (nm)	Stokes shift (cm^−1^)	Φ_f_^[^ ^[b]^, ^[c]^ ^]^ (%)	τ^[^ ^d^ ^]^ (ns)	*k*_r_^[^ ^e^ ^]^ (10^6^ s^−1^)	*k*_nr_^[^ ^f^ ^]^ (10^6^ s^−1^)	*g*_abs_ (*λ*) (10^−4^)	Average *g*_lum_ (10^−4^)	1PE *B*_CPL_^[^ ^g^ ^]^ (M^−1^ cm^−1^)
*^n^* Pr (**2a**)	533	14 610	580	1521	70	15.7	45	19	+1.7/−1.4 (572)	+1.0/−1.1	0.5
Me (**2b**)	531	14 280	580	1591	74	16.6	46	16	+0.8/−1.2 (554)	+2.1/‐2.3	1.0
*^i^* Pr (**2c**)	535	14 010	589	1714	75	17.0	44	15	+3.0/‐2.9 (543)	+4.8/‐4.9	2.6
*^c^* Hex (**2d**)	539	15 280	593	1690	72	16.5	44	17	+4.6/‐4.5 (548)	+5.8/‐6.7	3.4

^[a]^ Concentrations 1 × 10^−5^ to 5 × 10^−6^ M.

^[b]^ Φ_f_ estimated error = ±10%.

^[c]^ rhodamine B (Φ_f_ = 70% in MeOH).

^[d]^ *λ*_exc_ = 400 nm.

^[e]^ *k*_r_ = Φ_f_ /τ.

^[f]^ *k*_nr_ = (1 − Φ_f_)/τ.

^[g]^ *B*_CPL_ = ε × Φ_f_ ×|*g*_lum_|/2.^[^ ^15^ ^]^

**Figure 2:**
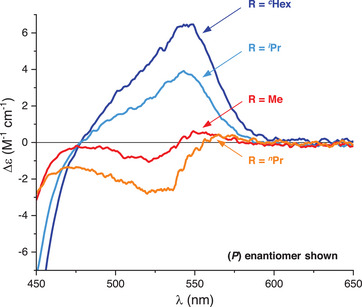
Comparison of ECD signatures at low energies of (*P*)‐**2a** (orange), (*P*)‐**2b** (red), (*P*)‐**2c** (light blue), and (*P*)‐**2d** (dark blue) in air‐equilibrated acetonitrile at RT between 450 and 650 nm and concentration 5 × 10^−5^ M.

ECD and CPL spectra were also recorded in acetonitrile and showed, for all derivatives, a mirror image relationship for pairs of (*M*) and (*P*) enantiomers. Compound **2b** (R = Me) shows similar ECD signatures to **2a** (R = *^n^* Pr) across the whole UV‐vis range, with pronounced Cotton effects in the UV domain and modest bands (|Δε| < 10 M^−1^ cm^−1^) above 400 nm (Figure S6 and S8), corresponding to |*g*_abs_| ≈ 1 × 10^−4^ for the lowest‐energy Cotton effect (Table 1). On the contrary, ECD features of derivatives bearing bulkier N‐substituents, **2c** and **2d**, are globally different from those of **2a** and **2b**. Looking across the whole UV‐vis range (Figures S10 and S12), aside from one strong peak below 300 nm (|Δε| ≈ 75 M^−1^ cm^−1^), the ECD signals remain < 25 M^−1^ cm^−1^. However, at low energies (> 500 nm), the branched N‐substituents provoke a higher chiroptical response in comparison to linear N‐alkyl chains. Similarly to what was observed from the unfunctionalized scaffolds,^[^ ^14^ ^]^ the *g*_abs_ is enhanced by a factor of 2 and 3 from **2a** to **2c** and **2d**, respectively (Table 1). The effect of sterically demanding N‐substituents (R = *^i^* Pr and *^c^* Hex) on the most red‐shifted Cotton effect is displayed clearly in Figure 2. The ECD features in this region are particularly sensitive to the nature of the substituents linked to the helicene core, as also evidenced in other helical systems.^[^ ^19^ ^]^ Derivatives **2a** and **2b** show, above 500 nm, an inversion of sign (negative → positive, for the (*P*) enantiomer) already reported for the introduction of *para*‐OMe moieties onto **1a**.^[^ ^4d^ ^]^ However, for **2c** and **2d**, this inversion is not present. Looking at the ECD of the (*P*) enantiomer (Figure 2), it can be hypothesized that the stronger positive chiroptical response induced by the branched N‐alkyl chains overrides the negative signature. Overall, all triple *para*‐OMe (*M*)‐**2** and (*P*)‐**2** enantiomers maintain negative and positive signs for the lowest‐energy ECD band, respectively. As expected, the same trend is observed in the emission of polarized light (Figures S7, S9, S11, and S13).^[^ ^20^ ^]^ *Para*‐OMe **2b** has low CPL similarly to **2a** (Table 1), while both isopropyl and cyclohexyl derivatives (**2c** and **2d**) exhibit higher average *g*_lum_ values, equal to + 4.8/‐4.9 × 10^−4^ and + 5.8/‐6.7 × 10^−4^, respectively. In previous work,^[^ ^4d^ ^]^ it was pointed out that, thanks to the strongly enhanced fluorescence of **2a**, the overall efficiency of circularly polarized emission (1PE *B*_CPL_, Table 1) is enhanced with respect to the unfunctionalized helicene **1a** (0.2 M^−1^ cm^−1^). Here, for derivatives **2c** and **2d**, in addition to the high Φ_f_, the dissymmetry of emission is also improved giving interesting *B*_CPL_ values of 2.6 and 3.4 M^−1^ cm^−1^, respectively. These values are among the highest obtained for cationic [n]helicenes.^[^ ^15^ ^]^

### 2.3 MP Excitation

**Figure 3:**
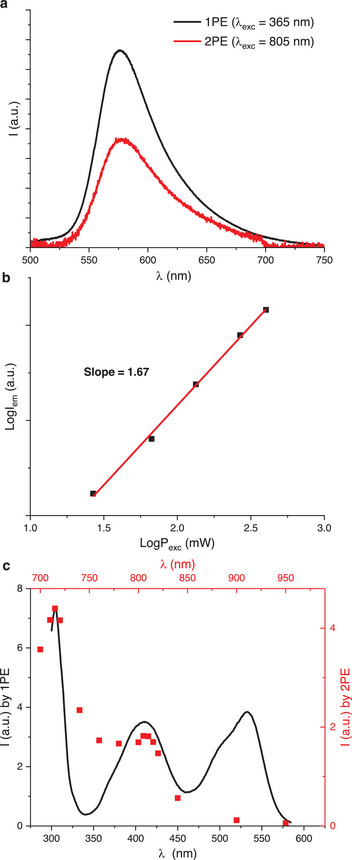
2PE studies of **2a** in acetonitrile with concentration 1 × 10^−5^ M. a) Emission spectra under 1PE (black line) and 2PE (red line); b) log versus log plot of the excitation‐power dependence of the emission intensity, the linear fit is displayed by a red line; c) 2PE (red dots, top x‐axis) and 1PE (black line, bottom axis) spectra followed at *λ*_em_ = 580 nm (x and y axes are adjusted to emphasize the agreement of the spectral features).

Initial 2PE studies were conducted with compound **2a** in acetonitrile. 2PE was carried out exciting the sample at 805 nm, using a tunable femtosecond pulsed laser (680–1300 nm, Coherent Discovery NX TPC, 100 fs, 80 MHz).^[^ ^13a^ ^]^ In Figure 3a are reported the emission profiles of **2a** obtained by 1PE and 2PE. Importantly, the same emission spectrum is obtained exciting in both the UV (365 nm) or in the NIR (805 nm). To confirm that a two‐photon process takes place while exciting at 805 nm, the emission intensity‐excitation power dependence was recorded and their logarithmic relationship was plotted. As anticipated, the LogI_em_ versus LogP_exc_ plot yielded a linear trend with slope 1.67 (Figure 3b), close to the theoretical value of 2.^[^ ^7b^ ^]^ The 2PE spectrum was also recorded and compared to that obtained by 1PE (Figure 3c). The 2PE spectrum of **2a** displays maxima at 715 and 805/810 nm, which follow the profile of the second and third one‐photon absorption bands. Oppositely, two‐photon absorption is not observed in correspondence to lowest‐energy S_0_–S_1_ transition. As expected because of the nonlinear effects of 2PE, the 2PE spectrum has not exactly double wavelengths compared to the 1PE spectrum.^[^ ^7^, ^13^, ^21^ ^]^

The ability of derivatives **2b**, **2c,** and **2d** to undergo 2PE was also characterized in the same manner; all related spectra are reported in the Supporting Information (Figures S16–S21). Plotting the emission intensity versus excitation power (*λ*_exc_ = 810 nm) in logarithmic scale for compounds **2b** to **2d**, slopes of around 2 were found for all the three curves, confirming again that the excitation process occurs via two‐photon absorption. 2PE spectra were also obtained. As for derivative **2a**, the one‐photon lowest energy absorption band is not active in 2PE, while the second and third bands appear in the 2PE spectra of all three compounds. The 2PE maxima are located at 750 and 810 nm (Figure S17), 780 and 810 nm, (Figure S19), and 770 and 810 nm (Figure S21) for **2b**, **2c,** and **2d**, respectively. The absorption band centered at 400 nm is the one that finds the best correspondence in 2PE, with all four fluorophores under study being active in 2PE at 810 nm. Values of 2PE CSs were then calculated at 810 nm using the established procedure and rhodamine *B* (σ^2^ at 810 nm = 260 GM in MeOH) as a reference (Table 2).^[^ ^10b^ ^]^ All triple *para*‐OMe diaza[4]helicenes are good two‐photon chromophores in the NIR, presenting CS values ≥ 100 GM. Overall, derivatives **2c** and **2d**, bearing N‐*^i^* Pr and N‐*^c^* Hex side chains, have the highest CS values.

**Table 2:** 2PE chiroptical properties of *para*‐OMe diaza[4]helicenes 2 in acetonitrile.

Compound^[^ ^a^ ^]^	σ^2^ at 810 nm (GM)^[^ ^b^ ^]^	Average *g*_lum_ (10^−4^)	2PE *B*_CPL_^[^ ^c^ ^]^ (GM)
*^n^* Pr (**2a**)	99	n.d.	n.d.
Me (**2b**)	106	n.d.	n.d.
*^i^* Pr (**2c**)	117	+6.6/‐6.1	0.03
*^c^* Hex (**2d**)	137	+9.4/‐9.2	0.05

^[a]^ Concentrations 1 × 10^−5^ M.

^[b]^ rhodamine B (Φ_f_ = 70% and σ^2^ = 260 GM in MeOH).

^[c]^ 2PE *B*_CPL_ = σ^2^ × Φ_f_ ×|*g*_lum_|/2.^[^ ^13a^ ^]^ n.d.: not determinable.

**Figure 4:**
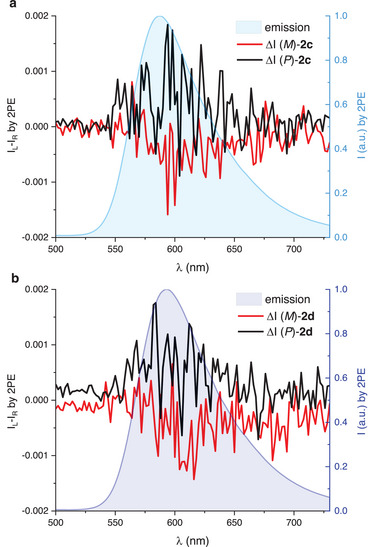
2PE CPL spectra (*λ*_exc_ = 810 nm) of compounds a) **2c** and b) **2d** in acetonitrile with concentrations of 1 × 10^−5^ M respectively.

At this point, for derivatives **2c** and **2d**, which combine favorable chiroptical response and higher values of CS, using the pulsed laser at 810 nm, 2PE CPL spectra were successfully acquired (Figure 4). The resulting 2PE CPL spectra are noisy due to the experimental challenges in recording the small signal deriving by the combination of 2PE and CPL emission (*g*_lum_ values < 10^−3^). Nonetheless, the allied 2PE CPL spectra of **2c** and **2d** well reproduce the 1PE CPL profiles (Figures S11 and S13) both in sign and in intensity. In fact, 2PE average *g*_lum_ values of + 6.6/‐6.1 × 10^−4^ and + 9.4/‐9.2 × 10^−4^ were found for **2c** (R = *^i^* Pr) and **2d** (R = *^c^* Hex), respectively. These values are comparable to those recorded using single photon excitation at shorter wavelengths (Table 1). Finally, 2PE *B*_CPL_ values can be calculated in the same fashion as the most classical 1PE *B*_CPL_, replacing the molar absorption coefficient with the 2PE CS value:^[^ ^13a^ ^]^

$$2\mathrm{PE}\;B_{\mathrm{CPL}}=\sigma^{2}\times \phi \times \left|g_{\mathrm{lum}}\right|/2.$$

The calculated 2PE *B*_CPL_ correspond to 0.03 and 0.05 GM for compounds **2c** and **2d**, respectively (Table 2). In comparison, recently reported chiral Eu complexes possess 2PE *B*_CPL_ values which are only three times higher (0.16/0.17 GM), even though presenting *g*_lum_ values of 10^−2^.^[^ ^13b^ ^]^ This is possible due to the overall favorable 2PE and emission properties (high Φ_f_) of *para*‐OMe derivatives **2** that compensate for the modest emission dissymmetry.

### 2.4 Microscopy

**Figure 5:**
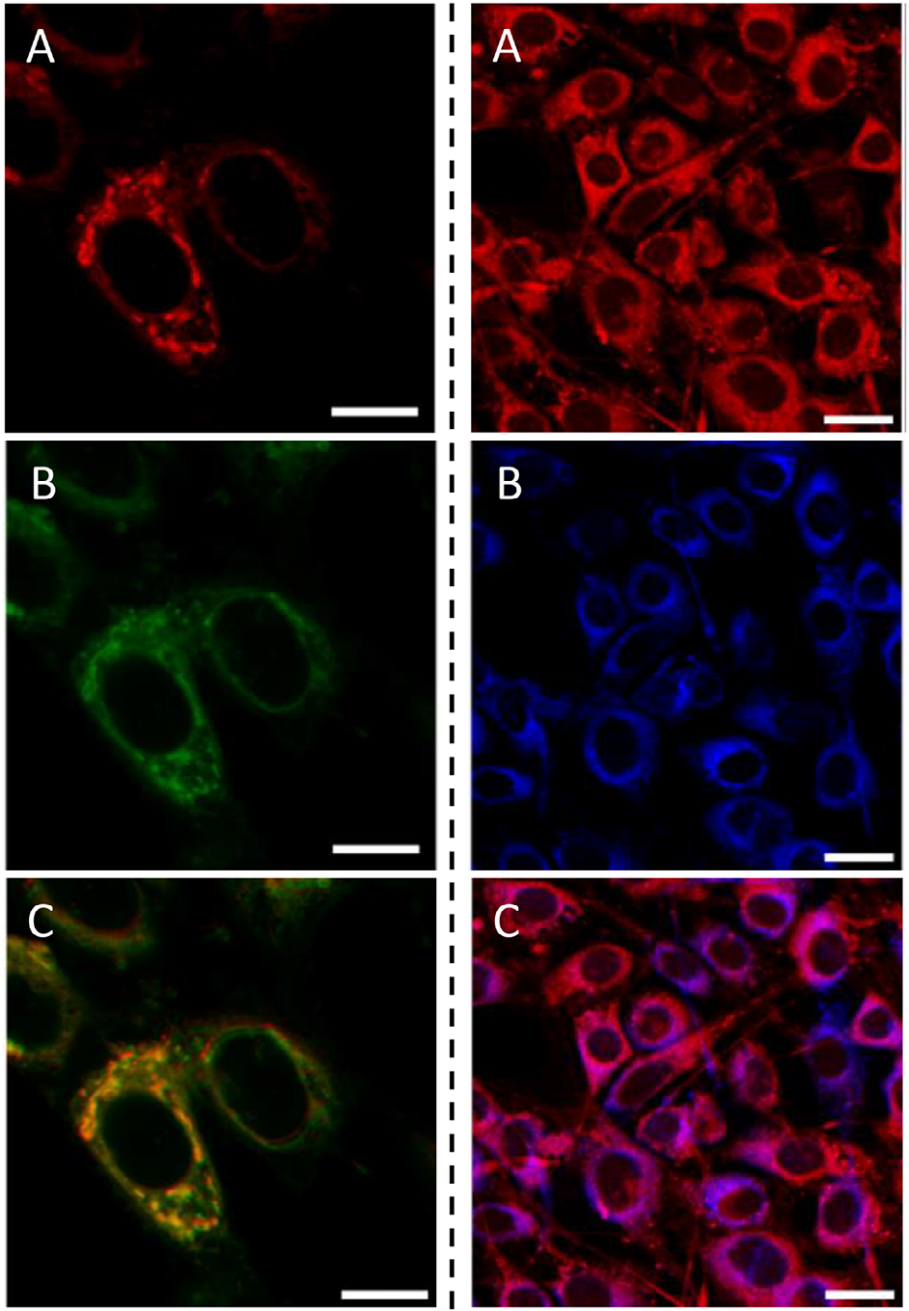
Live cell LSCM of mouse embryonic fibroblasts (NIH‐3T3). Left: A) compound **2d**, *λ*_exc_ = 543 nm, *λ*_em_ = 560–700 nm; B) ER‐Tracker™ Green, *λ*_exc_ = 488 nm, *λ*_em_ = 500–550 nm; C) RGB merge of A) and B) showing strong localization to the ER. Scale bar = 10 µm. Right: 2PE of live cell LSCM incubated with **2d** (500 nM) for 2 hours. A) *λ*_exc_ = 543 nm, *λ*_em_ = 560–700 nm. B) *λ*_exc_ = 810 nm, *λ*_em_ = 500–550 nm. C) RGB merge of A) and B) showing overlap of single and 2PE. Scale bar = 25 µm.

Live cell imaging was performed using laser scanning confocal microscopy (LSCM) to determine the two‐photon activation potential of the compounds and their localization. In practice, only compound **2d** was selected as it possesses the highest experimental two‐photon CS (137 GM) of all derivatives **2**. NIH‐3T3 (Swiss albino mouse embryonic fibroblasts) were incubated with 500 nM of **2d** for 2 hours. In Figure 5 (left), localization of **2d** in the endoplasmic reticulum (ER) is confirmed by co‐staining with ER‐Tracker Green (Invitrogen). The Pearson's coefficient for **2d** and ER‐Tracker Green is 0.89 and the Manders’ coefficients are 0.998 and 0.835 showing a strong localization to the ER. Since **2d** allies a favorable two‐photon CS and a high concentration in a small area in the live imaging, both these factors make it a good candidate for 2PE microscopy.^[^ ^13^, ^22^ ^]^ The local maximum of 810 nm was used to excite the compound rather than the overall maximum of 720 nm to allow for the full collection of the emission. Additionally, the output power of the laser is significantly higher at 810 nm than at 720 nm (∼3510 mW and ∼1530 mW, respectively), compensating the lower absorption intensity, whilst 810 nm wavelength is lower in energy and allows deeper tissue penetration in bioimaging. In addition, Figure 5 (right) shows single and two‐photon excitation images for **2d** and the merge of the two excitation modes. It is worth noting that an exact overlay is not achieved due to the slightly different focal (*z*) planes of the one and two‐photon excitation voxels because of the inherent chromatic aberrations of the LSCM system.

## 3 Conclusion

In this work, a family of triple *para*‐OMe cationic diaza[4]helicenes was prepared, by late‐stage functionalization of DMQA derivatives bearing linear and branched alkyl chains. Expanding from previously reported results,^[^ ^4d^ ^]^ *para*‐OMe groups were successfully introduced on the different helical scaffolds, showing the generality of the tandem borylation / hydroxylation procedure. While all *para*‐OMe derivatives **2** are strongly fluorescent with Φ_f_ ≈ 70%, higher *B*_CPL_ values of 2.6 and 3.4 M^−1^ cm^−1^ are obtained with compounds **2c** and **2d**, respectively. These values, which are among the highest obtained for cationic [n]helicenes,^[^ ^15^ ^]^ are possible, thanks to the combination of high Φ_f_ and the enhanced chiroptical properties arising with branched N‐substituents.^[^ ^14^ ^]^ Additionally, all *para*‐OMe derivatives revealed to be good two‐photon chromophores with CS values ≥ 100 GM at 810 nm. For compounds bearing branched N‐alkyl chains, 2PE CPL spectra were also successfully recorded giving emission profiles and *g*_lum_ values comparable to those of 1PE. Average 2PE *g*_lum_ values of + 6.6/‐6.1 × 10^−4^ and + 9.4/‐9.2 × 10^−4^ were found for **2c** (*^i^* Pr) and **2d** (*^c^* Hex), respectively, corresponding to 2PE *B*_CPL_ of 0.03 and 0.05 GM. Noteworthy, acquisition of 2PE CPL for small organic molecules is rather unique. Finally, test studies of **2d** in cell imaging showed the viability of compound class **2** in 1PE and 2PE confocal microscopy with localization in the ER.

## Supporting Information

It contains general methods, experimental conditions, full characterization of new compounds, (chir)optical measurements, time‐correlated single photon counting, additional 2PE data, CSP‐HPLC traces and NMR spectra. The authors have cited additional references within the Supporting Information.^[^ ^23^ ^]^

## Conflict of Interests

The authors declare no conflict of interest.

## Supporting information



Supporting Information

## Data Availability

The data that support the findings of this study are openly available in yareta.unige.ch at https://doi.org/10.26037/yareta:bcq6hrszpjgy3naore3vz2ohwi. It will be preserved for 10 years.
