# Supplementary material for: Two‐photon Excitation of Bright Diaza[4]Helicenes for Isotropic and Circularly Polarized Emission
Source: Chemistry. 2025 May 2;31(32):e202501212. doi: 10.1002/chem.202501212 (PMC12144873; doi:10.1002/chem.202501212)
Supplement: Supplementary file 1 — Supporting Information [file CHEM-31-e202501212-s001.pdf]

Two-photon excitation of bright diaza[4]helicenes for isotropic and circularly polarized emission

Bibiana Fabri,[a] Davide F. De Rosa,[b] Dominic J. Black,[b] Rebecca Mucci,[a] Artemijs Krimovs,[b] Robert Pal,*[b] Jérôme Lacour*[a]

[a] Bibiana Fabri, Rebecca Mucci, Jérôme Lacour – Department of Organic Chemistry, University of Geneva, Quai Ernest Ansermet 30, 1211 Geneva 4 (Switzerland) E-mail: jerome.lacour@unige.ch

[b] Davide F. De Rosa, Dominic J. Black, Artemijs Krimovs, Robert Pal – Department of Chemistry, Durham University, Durham DH1 3LE, U.K.; Email: robert.pal@durham.ac.uk

Supporting Information

Contents

General remarks and analysis conditions S3

Synthesis and characterization of new compounds S7

3,7,11-trihydroxy-1,13-dimethoxy-5,9-dimethyl-5,9-dihydro-13bH-quinolino[2,3,4-kl]acridin-13b-ylium hexafluorophosphate salt (**4b**) S7

3,7,11-trihydroxy-5,9-diisopropyl-1,13-dimethoxy-5,9-dihydro-13bH-quinolino[2,3,4-kl]acridin-13b-ylium hexafluorophosphate salt (**4c**) S7

5,9-dicyclohexyl-3,7,11-trihydroxy-1,13-dimethoxy-5,9-dihydro-13bH-quinolino[2,3,4-kl]acridin-13b-ylium hexafluorophosphate salt (**4d**) S8

1,3,7,11,13-pentamethoxy-5,9-dimethyl-5,9-dihydro-13bH-quinolino[2,3,4-kl]acridin-13b-ylium hexafluorophosphate salt (**2b**) S8

5,9-diisopropyl-1,3,7,11,13-pentamethoxy-5,9-dihydro-13bH-quinolino[2,3,4-kl]acridin-13b-ylium hexafluorophosphate salt (**2c**) S9

5,9-dicyclohexyl-1,3,7,11,13-pentamethoxy-5,9-dihydro-13bH-quinolino[2,3,4-kl]acridin-13b-ylium hexafluorophosphate salt (**2d**) S9

Chiral Stationary Phase (CSP) HPLC S11

Enantiomeric excess analysis of **2a** S11

Resolution of **2b** S12

Resolution of **2c** S13

Resolution of **2d** S14

(Chir)Optical properties S15

Absorption and emission spectra of compounds **2** S15

ECD and CPL spectra of compounds **2a** S15

ECD and CPL spectra of compounds **2b** S17

ECD and CPL spectra of compounds **2c** S18

ECD and CPL spectra of compounds **2d** S20

Lifetime measurements S21

Additional two-photon excitation data S22

$LogI_{em}$ *vs* $LogP_{exc}$ plot of **2a** with fitting data S22

2PE measurements of **2b** S22

2PE measurements of **2c** S23

2PE measurements of **2d** S24

^{1}H, ^{13}C, ^{19}F NMR spectra, IR spectra and HRMS reports of new compounds S26

Compound **4b** S26

Compound **4c** S30

Compound **4d** S34

Compound **2b** S38

Compound **2c** S42

Compound **2d** S46
References S50

General remarks and analysis conditions

Dataset

The dataset for this article can be found at the following DOI: 10.26037/yareta:bcq6hrszpjgy3naore3vz2ohwi. It will be preserved for 10 years.

Reagents and solvents: Unless otherwise stated, reagents were purchased from commercial sources and used without further purification. All reactions involving air sensitive compounds were carried out under N_2 *via* an inert gas/vacuum double manifold line and standard Schlenk techniques using dry solvents. Reactions involving oxygen sensitive reagents were performed using degassed solvents. Tetrahydrofuran (THF) was distilled under N_2 atmosphere over sodium and benzophenone.

Chromatography: **Analytical thin layer chromatography** (TLC) and **retardation factors** (R_f) were performed with Silica gel 60 F_{254} aluminium plates purchased from Merck. **Flash column chromatograph** was performed with Silica SiliaFlash P60, 40-63 µm (230-400 mesh) and with Combi*Flash*® *R*f 200 on SiO_2 4 g, 12 g and 24 g cartridges. **Chiral stationary phase (CSP) HPLC** to obtain enantiopure **2b**, **2c** and **2d** was performed on an Agilent 1260 Infinity II apparatus (quaternary pump, auto sampler, column thermostat and diode array detector) using a semi-preparative CHIRALPAK® IC column (250 x 10 mm, 5 µm) and HPLC grade solvents.

Nuclear Magnetic Resonance: NMR spectra were recorded on a Bruker Avance III 500 MHz, Bruker Avance III HD-*NanoBay* 400 MHz and Bruker Avance III HD-*NanoBay* 300 MHz spectrometers at room temperature. **^{1}H NMR** chemical shifts are given in ppm relative to Me_4Si with solvent resonances used as internal standards (CD_2Cl_2 δ = 5.32 ppm). Data are reported as follows: chemical shift (ppm) on the *δ* scale, multiplicity, coupling constant (Hz) and integration. **^{13}C NMR** chemicals shifts are given in ppm relative to Me_4Si with solvent resonances used as internal standards (CD_2Cl_2 δ = 53.84 ppm). **^{19}F NMR** chemicals shifts are given in ppm.

Infrared Spectroscopy: IR spectra were recorded with a Perkin-Elmer 100 FT-IR spectrometer using a diamond ATR Golden Gate sampling and are reported in wavenumbers (cm^{-1}).

Melting points were measured on a standard melting point apparatus in open capillary vials and are uncorrected.

Mass Spectrometry: LRMS spectra were obtained in methanol solutions on an API 150EX (AB/MDS Sciex) spectrometer in positive polarity. **HRMS** spectra were obtained in methanol solutions on a Waters Xevo G2 Tof (TOF) spectrometer in positive polarity by the Department of Mass Spectroscopy at the University of Geneva.

(Chir)Optical properties: all (chir)optical measurements were performed in 1 cm optical path quartz cells, unless otherwise stated.

Optical rotations (OR) were measured on a Perkin Elmer 241 polarimeter at room temperature using a Hg lamp (365 nm) in a 1 dm optical quartz cell. All compounds under study in this work absorb strongly at wavelength used to measure the optical rotation (365 nm), therefore the reported OR values are only indicative as they might be affected from CD effects. **UV-Vis-NIR absorption spectra** were recorded on a JASCO V-650 spectrophotometer at room temperature. Measurements were performed in air-equilibrated analytical grade acetonitrile at concentrations *ca.* 10^{-5} M. **Electronic Circular Dichroism (ECD)** spectra were recorded on a JASCO J-815 spectrophotometer at room temperature with parameters as follows: scan speed – 200 nm/min, slit width – 1 nm, integration time – 1 sec, multiple accumulations. Measurements were performed in air-equilibrated analytical grade acetonitrile at concentrations *ca.* 1 or 5 $\cdot 10^{-5}$ M. All spectra were baseline corrected by subtraction of the solvent spectrum. **Steady-state fluorescence** spectra were measured using a FluoroMax+ spectrofluorometer from Horiba Scientific. All fluorescence spectra were corrected for the wavelength-dependent sensitivity of the detection. Fluorescence quantum yields ϕ_f were determined by comparison with a standard of known quantum yield using the following equation:

$$\Phi = \Phi_r \frac{I\ A_r\ n^2}{I_r\ A\ n_r^2}$$

where *A* is the absorbance at the excitation wavelength (λ), *n* the refractive index and *I* the integrated emission intensity; "r" stands for reference. Diluted solutions with absorption lower than 0.1 were employed. Excitations of reference and sample compounds were performed at the same wavelength. **Fluorescence lifetimes** on the nanosecond timescale were measured by a time-correlated single photon counting (TCSPC) setup. Excitation was performed at 400 nm using ~60 ps pulse at 20 MHz produced by a laser diode (PicoQuant, LDH-P-C-400). The fluorescence decay was followed at wavelength 610 nm for all derivatives using band-pass filters. The full width at half-maximum (fwhm) of the instrument response function (IRF) was around 200 ps. The fluorescence time profiles were analysed with the deconvolution of the experimental IRF and an exponential function.

CPL spectroscopy

PEM-CPL spectrometer: CPL was measured with a home-built (modular) spectrometer.[1] The excitation source was a broad band (200 – 1000 nm) laser-driven light source EQ 99 (Elliot Scientific). The excitation wavelength was selected by feeding the broadband light into an Acton SP-2155 monochromator (Princeton Instruments); the collimated light was focused into the sample cell (1 cm quartz cuvette). Sample PL emission was collected perpendicular to the excitation direction with a lens (f = 150 mm). The emission was fed through a photoelastic modulator (PEM) (Hinds Series II/FS42AA) and through a linear sheet polariser (Comar). The light was then focused into a second scanning monochromator (Acton SP-2155) and subsequently on to a photomultiplier tube (PMT) (Hamamatsu H10723 series). The detection of the CPL signal was achieved using the field modulation lock-in technique. The electronic signal from the PMT was fed into a lock-in amplifier (Hinds Instruments Signaloc Model 2100). The reference signal for the lock-in detection was provided by the PEM control unit. The monchromators, PEM control unit and lock-in amplifier were interfaced to a desktop PC and controlled by a custom-written Labview graphic user interface. The lock-in amplifier provided two signals, an AC signal corresponding to (I_L- I_R) and a DC signal corresponding to (I_L + I_R) after background subtraction. The emission dissymmetry factor was therefore readily obtained from the experimental data, as 2 AC/DC. Spectral calibration of the scanning monochromator was performed using an Hg-Ar calibration lamp (Ocean Optic, HG-2) A correction factor for the wavelength dependence of the detection system was constructed using a calibrated lamp (Ocean Optics, CAL-2000). The measured raw data was subsequently corrected using this correction factor. The validation of the CPL detection systems was achieved using light emitting diodes (LEDs) at various emission wavelengths. The LED was mounted in the sample holder and the light from the LED was fed through a broad band polarising filter and $\lambda/4$ plate (Ocean Optics) to generate circularly polarised light. Prior to all measurements, the $\lambda/4$ plate and a LED were used to set the phase of the lock-in amplifier correctly. The emission spectra were recorded with 2 nm/ 1 ms step size and the slits of the detection monochromator were set to 5 nm width corresponding to a spectral resolution of 2 nm. CPL spectra (as well as total emission spectra) were obtained through an averaging procedure of 20 scans. All the spectra were recorded in air-equilibrated analytical grade acetonitrile at concentrations *ca*. 10^{-5} M, using a standard 1 cm path length quartz cuvette.

MP-Spectroscopy

Two photon excitation CPL spectroscopy has been achieved by coupling (beam routing using mirrors, Thor Labs BB1-E03) a tunable femtosecond pulsed laser (680 – 1300 nm, Coherent Discovery TPC, 100 fs, 80 MHz) to two pre-existing CPL spectrometers detailed above. Initial proof of concept two photon spectroscopy has been achieved by perpendicularly mounting an Ocean Optics HR2000Pro (2048-pixel linear CCD Sony ILX5 chip, 200 µm slit, H3 grating, 350 – 850 nm spectral region) spectrometer as a 'third arm' to the Discovery TPC laser. The laser beam was focused onto the centre of the 1cm path sample holder (Thor labs CVH100) by a dedicated ultrafast laser lens (Edmund Optics 11711, 50 mm focal length). The spectrometer has also been equipped with a perpendicularly mounted 365 nm LED (nichia, 1W) and been operated using a modified version of the above-mentioned custom time resolved detection and accumulation algorithm written in Labview2013 program. In order to eliminate unwanted artefacts associated with stray light from MP excitation each spectrometer have been equipped with a rotating filter wheel (Thor Labs, CFW6) housing an LP420 (Comar Optics, for 365 nm UVLED excitation) and SP650 – SP700, 25 nm spectral intervals (Edmund Optics, for MP excitation) filters.

MP-CPL spectra were obtained using the following parameters: slits – 9 nm max, integration time – 1 ms, resolution step – 2 nm, accumulations – 20.

Cross section determination

The cross-sections (σ^2) of the compounds discussed herein are calculated according to established procedures:[2]

$$\sigma_s^2 = \sigma_r^2 \cdot \Phi_r \cdot \frac{C_r n_s F_s(\lambda)}{\Phi_s C_s n_r F_r(\lambda)}$$

Where s is sample, *r* is reference, ϕ is the total emission quantum yield of the compound, *C* is the concentration, *n* the refractive index and $F_s(\lambda)$ and $F_r(\lambda)$ are the integrated PL spectrum for the sample and reference, respectively. Additionally, we have demonstrated that the excitation process is definitely a two-photon event by recording an excitation power dependence; the resulting line has a slope of two on a logarithmic scale.[3] The cross sections were calculated with reference to rhodamine B in methanol. The MP spectra were recorded in 1 cm path length quartz cuvettes, in air-equilibrated analytical grade acetonitrile at concentrations *ca*. 10^{-5} M.

In the reaction conditions, RT indicates an ambient temperature of 20 to 30 °C.

Live cell cultures studies

CPL - Laser scanning confocal microscopy experiments were conducted using NIH 3T3 embryonic mouse skin fibroblast cell line, sourced from ATCC (CRL-1658) and were established and maintained in a category 2 cell culture facility according to established standardized protocol for 12 months; they were periodically monitored for mycoplasma contamination.[4] Cells were maintained in exponential growth as monolayers in F-12/DMEM (Dulbecco's Modified Eagle Medium) 1:1 that was supplemented with 10% fetal bovine serum (FBS). Cells were grown in 75 cm^2 plastic culture flasks, with no prior surface treatment. Cultures were incubated at 37 °C, 10% average humidity and 5% (v/v) CO_2. Cells were harvested by treatment with 0.25% (v/v) trypsin solution for 5 min at 37 ºC. Cell suspensions were pelleted by centrifugation at 1000 rpm for 3 min, and were re-suspended in fresh medium by repeated aspiration with a sterile plastic pipette. Microscopy cells were seeded in untreated iBibi 100 µL live cell channels and allowed to grow to 40% to 60% confluence, at 37 °C in 5% CO_2. At this stage, the medium was replaced and cells were treated with the studied nanomachines and co-stains as appropriate, with 0.1 % DMSO (as detailed above) present in the final imaging medium. For live cell imaging, DMEM/F12 media (10% FBS) lacking phenol red was used from this point onwards. Following incubation, where Method B was used, the channels were washed with live cell imaging media and imaged using a purpose build incubator housing the microscope maintaining 37 °C, 5% CO_2 and 10% humidity.

Steady state fluorescence microscopy

1PE Microscopy

Steady state fluorescence images were recorded using a PhMoNa[5] enhanced Leica SP5 II LSCM confocal microscope equipped with a HCX PL APO 63x/1.40 NA LambdaBlue Oil immersion objective. Data were collected using 5x digital magnification at 400 Hz/line scan speed (4 line average, bidirectional scanning) at 458 nm Ar laser) with 3 mW laser power emission collected between 550 – 730 nm. In order to achieve excitation with maximal probe emission, the microscope was equipped with a triple channel imaging detector, comprising two conventional PMT systems and a HyD hybrid avalanche photodiode detector. The latter part of the detection system, when operated in the BrightRed mode, is capable of improving imaging sensitivity by 25%, reducing signal to noise by a factor of 5. Frame size was determined at 2048 x 2048 pixel, with 0.6 airy disc unit determining the applied pinhole diameter rendering on voxel to be corresponding to 24.02 x 24.02 nm (frame size 49.16 x 49.16 µm) with a section thickness of 380 nm. An Ar ion laser was used when 488 nm, 2mW, emission collected 500-530 nm) were used to corroborate cellular compartmentalization.

All post image processing was carried out on the open source, plugin prepacked, FIJI (ImageJ 1.52p Java 1.8.0_172 64 Bit).[6] All adjustments to voxel brightness and contrast were kept at constant values within each

image set. Manders' overlap coefficient (MOC) and Pearson's correlation coefficient (PCC) values were calculated using the JACoP v2.1.4 plugin for imageJ.[7]

Multiphoton Microscopy

Multiphoton microscopy has been conducted by coupling our a Leica SP5 to a Coherent Chameleon Vision tunable (680 – 1080 nm, 65 mW @ 810 nm, 80 MHz, 100 fs) multiphoton using a x20 0.7NA oil objective (operating at 100 Hz scan speed with 2 line accumulation uni-directional 1024 × 1024 pixel FOV. Probe emission collected at 550-730 nm.

Synthesis and characterization of new compounds

Racemic **1a**, **1b**, **1c**, **1d** were synthetized according to the reported procedures.[8]

Racemic **2a** and its (*M*) and (*P*) enantiomers were synthetized as previously reported.[9]

Tris(OH) derivatives **4** are used as synthetic intermediates only and have not been employed for photophysical and biological studies. Isolated yields are slightly approximate as samples may include small impurities such as residual solvents and grease traces (*vide infra*).

3,7,11-trihydroxy-1,13-dimethoxy-5,9-dimethyl-5,9-dihydro-13bH-quinolino[2,3,4-kl]acridin-13b-ylium hexafluorophosphate salt (**4b**)

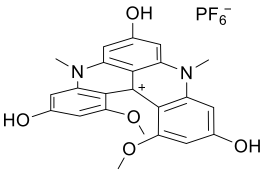

rac

Compound **4b** is synthetized following the same procedure reported for **4a** employing DMQA **1b** as starting material:[9]

(1): Preparation of intermediate **3b** using **1b** (50 mg, 0.11 mmol, 1 equiv).

(2) NaOH (solid, 4.4 mg, 0.11 mmol, 1 equiv), hydrogen peroxide 50% (63 µL, 1.1 mmol, 10 equiv) were used. The crude product is purified by flash chromatography (Combi*Flash*®, 12 g SiO_2 column, CH_2Cl_2/MeOH gradient, up to 4% MeOH. TLC CH_2Cl_2:MeOH = 9:1, R_f 0.2). Compound **4b** is obtained as a red solid (52.4 mg, 83%).

^{1}H NMR (500 MHz, MeOD) δ 6.71 (s, 2H, CH), 6.61 (d, *J* = 2.0 Hz, 2H, CH), 6.40 (d, *J* = 1.2 Hz, 2H, CH), 3.84 (s, 6H, OCH_3), 3.72 (s, 6H, NCH_3). **^{13}C NMR (126 MHz, MeOD)** δ 166.64 (C), 165.75 (C), 163.01 (C), 146.03 (C), 142.74 (C), 141.47 (C+), 114.03 (C), 108.10 (C), 95.28 (CH), 94.32 (CH), 92.93 (CH), 56.11 (OCH_3), 37.00 (NCH_3). **^{19}F NMR (282 MHz, MeOD)** δ -75.12 (d, *J* = 707.0 Hz, PF_6). **IR (neat, cm^{-1}):** ν = 2937.43, 1589.25, 1574.45, 1557.19, 1470.51, 1446.63, 1394.22, 1341.96, 1246.96, 1197.83, 1158.71, 1050.63, 1124.87, 1071.30, 946.41. **HRMS (ESI)** calculated for [M+] 405.1446, found 405.1449. **m.p.** 276 °C (degradation).

3,7,11-trihydroxy-5,9-diisopropyl-1,13-dimethoxy-5,9-dihydro-13bH-quinolino[2,3,4-kl]acridin-13b-ylium hexafluorophosphate salt (**4c**)

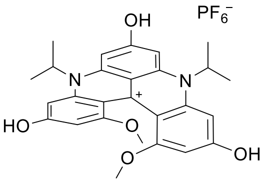

rac

Compound **4c** is synthetized following the same procedure reported for **4a** employing DMQA **1c** as starting material:[9]

(1): Preparation of intermediate **3c** using **1c** (50 mg, 0.1 mmol, 1 equiv).

(2) NaOH (solid, 4.0 mg, 0.1 mmol, 1 equiv), hydrogen peroxide 50% (58 µL, 1 mmol, 10 equiv) were used. The crude product is purified by flash chromatography (Combi*Flash*®, 12 g SiO_2 column, CH_2Cl_2/MeOH gradient, up to 4% MeOH. TLC CH_2Cl_2:MeOH = 9:1, R_f 0.3). Compound **4c** is obtained as a red solid (51.6 mg, 85%).

^{1}H NMR (500 MHz, MeOD) δ 6.95 (s, 2H, CH), 6.81 (d, *J* = 2.0 Hz, 2H, CH), 6.40 (d, *J* = 1.7 Hz, 2H, CH), 5.07 (hept, *J* = 7.0 Hz, 2H, NCH), 3.73 (s, 6H, OCH_3), 1.84 (d, *J* = 7.0 Hz, 6H, CH_3), 1.77 (d, *J* = 7.0 Hz, 6H, CH_3). **^{13}C NMR (126 MHz, MeOD)** δ 166.05 (C), 164.58 (C), 162.55 (C), 145.44 (C), 141.86 (C+), 141.49 (C), 116.57 (C), 109.75 (C), 96.33 (CH), 95.43 (CH), 94.33 (CH), 56.36 (NCH), 56.13 (OCH_3), 20.49 (CH_3), 19.79 (CH_3). **^{19}F NMR (282 MHz, MeOD)** δ -74.86 (d, *J* = 707.1 Hz, PF_6). **IR (neat, cm^{-1}):** ν = 3515.76, 3210.30, 2923.13, 2852.40, 2322.95, 2050.26, 1979.87, 1580.09, 1537.06, 1523.46, 1461.84, 1395.93, 1376.38, 1339.73, 1267.34, 1196.25, 1166.29, 1100.01, 992.39, 973.31. **HRMS (ESI)** calculated for [M+] 461.2071, found 461.2080. **m.p.** 247 °C (degradation).

5,9-dicyclohexyl-3,7,11-trihydroxy-1,13-dimethoxy-5,9-dihydro-13bH-quinolino[2,3,4-kl]acridin-13b-ylium hexafluorophosphate salt (**4d**)

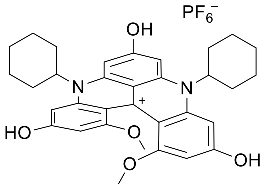

Compound **4d** is synthetized following the same procedure reported for **4a** employing DMQA **1d** as starting material:[9]

(1): Preparation of intermediate **3d** using **1d** (50 mg, 0.09 mmol, 1 equiv).

(2) NaOH (solid, 3.5 mg, 0.09 mmol, 1 equiv), hydrogen peroxide 50% (50 µL, 0.9 mmol, 10 equiv) were used. The crude product is purified by flash chromatography (Combi*Flash*®, 12 g SiO_2 column, CH_2Cl_2/MeOH gradient, up to 4% MeOH. TLC CH_2Cl_2:MeOH = 9:1, R_f 0.4). Compound **4d** is obtained as a red solid (55.6 mg, 90%).

^{1}H NMR (500 MHz, MeOD) δ 7.00 (s, 2H, CH), 6.86 (d, *J* = 2.0 Hz, 2H, CH), 6.39 (d, *J* = 1.7 Hz, 2H, CH), 4.60 – 4.44 (m, 2H, NCH)), 3.71 (s, 6H, OCH_3), 2.72 – 2.55 (m, 4H, CH_2), 2.12 (d, *J* = 12.8 Hz, 2H, CH_2), 2.09 – 2.00 (m, 4H, CH_2), 1.92 (d, *J* = 11.6 Hz, 2H, CH_2), 1.86 (d, *J* = 13.3 Hz, 2H, CH_2), 1.71 – 1.56 (m, 4H, CH_2), 1.53 – 1.40 (m, 2H, CH_2). **^{13}C NMR (126 MHz, MeOD)** δ 165.93 (C), 164.15 (C), 162.43 (C), 145.85 (C), 141.96 (C+), 141.67 (C), 116.87 (C), 109.96 (C), 96.69 (CH), 95.49 (CH), 94.41 (CH), 66.51 (NCH), 56.15 (OCH_3), 31.08 (CH_2), 30.14 (CH_2), 27.50 (CH_2), 27.47 (CH_2), 26.54 (CH_2). **^{19}F NMR (282 MHz, MeOD)** δ -74.74 (d, *J* = 707.7 Hz, PF_6). **IR (neat, cm^{-1}):** ν = 3119.33, 2934.05, 2854.00, 2162.67, 2050.16, 1979.59, 1578.93, 1473.32, 1453.54, 1389.76, 1338.46, 1271.53, 1251.31, 1197.68, 1163.74, 1132.00, 1101.02, 1053.86, 1007.24, 985.94, 943.33. **HRMS (ESI)** calculated for [M+] 541.2698, found 541.2718. **m.p.** 276 °C (degradation).

1,3,7,11,13-pentamethoxy-5,9-dimethyl-5,9-dihydro-13bH-quinolino[2,3,4-kl]acridin-13b-ylium hexafluorophosphate salt (**2b**)

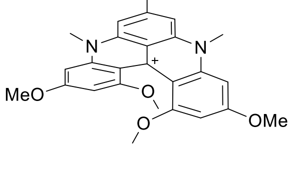

Compound **2b** is synthetized following the same procedure reported for **2a**:[9] **4b** (50 mg, 0.09 mmol, 1 equiv), methyl iodide (28 µL, 0.45 mmol, 5 equiv) and Cs_2CO_3 (102.6 mg, 0.31 mmol, 3.5 equiv) and DMF (4 mL) were used. The crude product is purified by flash chromatography (Combi*Flash*®, 12 g SiO_2 column, CH_2Cl_2/MeOH gradient, up to 2% MeOH. TLC CH_2Cl_2:MeOH = 98:2, R_f 0.11). Finally, further purification by precipitation with Et_2O and/or pentane is performed. Compound **2b** is obtained as a red solid (21.3 mg, 40%).

^{1}**H NMR (500 MHz, CD_2Cl_2)** δ 6.79 (s, 2H, CH), 6.62 (d, *J* = 2.1 Hz, 2H, CH), 6.43 (d, *J* = 2.1 Hz, 2H, CH), 4.11 (s, 3H, OCH_3), 4.07 (s, 6H. OCH_3), 3.99 (s, 6H, OCH_3), 3.75 (s, 6H, NCH_3). ^{13}**C NMR (126 MHz, CD_2Cl_2)** δ 167.25 (C), 166.18 (C), 161.62 (C), 145.30 (C), 141.89 (C), 140.91 (C+), 114.62 (C), 108.59 (C), 94.62 (CH), 92.76 (CH), 90.26 (CH), 57.01 (OCH_3), 56.80 (OCH_3), 56.29 (OCH_3), 37.72 (NCH_3). ^{19}**F NMR (282 MHz, CD_2Cl_2)** δ -73.35 (d, *J* = 710.3 Hz, PF_6). **IR (neat, cm^{-1}):** ν = 2995.70, 2923.72, 2851.06, 2632.66, 1602.11, 1580.77, 1574.38, 1504.56, 1461.74, 1454.62, 1434.54, 1418.00, 1387.39, 1348.56, 1331.45, 1317.22, 1302.11, 1279.97, 1257.49, 1237.22, 1213.18, 1180.15, 1156.73, 1125.84, 1057.61, 1042.07, 1000.06, 982.87, 956.41, 941.67. **HRMS (ESI)** calculated for [M+] 447.1915, found 447.1935. **m.p.** 296 °C (degradation).

To obtain (*M*) and (*P*) enantiomers, resolution of **2b** was performed with CSP-HPLC (*vide infra*). **OR** (MeCN) (*M*) $[\alpha]_{365}$ –3200 and (*P*) $[\alpha]_{365}$ +3704.

5,9-diisopropyl-1,3,7,11,13-pentamethoxy-5,9-dihydro-13bH-quinolino[2,3,4-kl]acridin-13b-ylium hexafluorophosphate salt (**2c**)

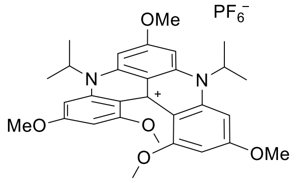

Compound **2c** is synthetized following the same procedure reported for **2a**:[9] **4c** (50 mg, 0.08 mmol, 1 equiv), methyl iodide (25 µL, 0.4 mmol, 5 equiv) and Cs_2CO_3 (91.2 mg, 0.28 mmol, 3.5 equiv) and DMF (4 mL) were used. The crude product is purified by flash chromatography (Combi*Flash*®, 12 g SiO_2 column, CH_2Cl_2/MeOH gradient, up to 2% MeOH. TLC CH_2Cl_2:MeOH = 98:2, R_f 0.14). Finally, further purification by precipitation with Et_2O and/or pentane is performed. Compound **2c** is obtained as a red solid (26.5 mg, 51%).

^{1}**H NMR (500 MHz, CD_2Cl_2)** δ 6.95 (s, 2H, CH), 6.77 (d, *J* = 2.1 Hz, 2H, CH), 6.42 (d, *J* = 1.9 Hz, 2H, CH), 5.12 (hept, *J* = 7.1 Hz, 2H, NCH), 4.09 (s, 3H, OCH_3), 4.05 (s, 6H, OCH_3), 3.73 (s, 6H, OCH_3), 1.91 (d, *J* = 7.1 Hz, 6H, CH_3), 1.82 (d, *J* = 7.0 Hz, 6H, CH_3). ^{13}**C NMR (126 MHz, CD_2Cl_2)** δ 166.52 (C), 164.66 (C), 161.29 (C), 144.71 (C), 141.35 (C+), 140.61 (C), 117.02 (C), 110.21 (C), 94.43 (CH), 94.32 (CH), 91.68 (CH), 56.78 (OCH_3), 56.63 (OCH_3), 56.26 (OCH_3), 56.10 (NCH), 20.92 (CH_3), 20.24 (CH_3). ^{19}**F NMR (282 MHz, CD_2Cl_2)** δ -73.50 (d, *J* = 710.3 Hz, PF_6). **IR (neat, cm^{-1}):** ν = 2925.64, 2852.82, 2162.14, 2049.69, 1979.61, 1736.27, 1601.54, 1575.65, 1511.72, 1462.25, 1427.66, 1401.56, 1376.08, 1335.42, 1285.14, 1268.69, 1252.91, 1209.02, 1164.95, 1101.96, 1057.69, 1010.91, 988.21, 946.58. **HRMS (ESI)** calculated for [M+] 503.2541, found 503.2537. **m.p.** 208 °C (degradation).

To obtain (*M*) and (*P*) enantiomers, resolution of **2c** was performed with CSP-HPLC (*vide infra*). **OR** (MeCN) (*M*) $[\alpha]_{365}$ –536 and (*P*) $[\alpha]_{365}$ +667.

5,9-dicyclohexyl-1,3,7,11,13-pentamethoxy-5,9-dihydro-13bH-quinolino[2,3,4-kl]acridin-13b-ylium hexafluorophosphate salt (**2d**)

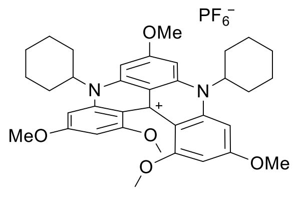

Compound **2d** is synthetized following the same procedure reported for **2a**:[9] **4d** (50 mg, 0.07 mmol, 1 equiv), methyl iodide (22 µL, 0.35 mmol, 5 equiv) and Cs_2CO_3 (81.5 mg, 0.25 mmol, 3.5 equiv) and DMF (4 mL) were used. The crude product is purified by flash chromatography (Combi*Flash*®, 12 g SiO_2 column, CH_2Cl_2/MeOH

gradient, up to 2% MeOH. TLC CH_2Cl_2:MeOH = 98:2, R_f 0.16). Finally, further purification by precipitation with Et_2O and/or pentane is performed. Compound **2d** is obtained as a red solid (49.1 mg, 92%).

^{1}H NMR (500 MHz, CD_2Cl_2) δ 7.00 (s, 2H, CH), 6.80 (d, *J* = 2.1 Hz, 2H, CH), 6.42 (d, *J* = 2.0 Hz, 2H, CH), 4.58 (tt, *J* = 12.6, 3.6 Hz, 2H, NCH), 4.09 (s, 3H, OCH_3), 4.05 (s, 6H, OCH_3), 3.74 (s, 6H, OCH_3), 2.60 (pd, *J* = 12.5, 3.8 Hz, 4H, CH_2), 2.24 (d, *J* = 13.1 Hz, 2H, CH_2), 2.13 – 2.02 (m, 4H, CH_2), 1.99 (d, *J* = 13.1 Hz, 2H, CH_2), 1.90 (d, *J* = 13.2 Hz, 2H, CH_2), 1.66 – 1.55 (m, 4H, CH_2), 1.41 (qt, *J* = 13.2, 3.6 Hz, 2H, CH_2). **^{13}C NMR (126 MHz, CD_2Cl_2)** δ 166.43 (C), 164.38 (C), 161.22 (C), 145.14 (C), 141.45 (C+), 140.88 (C), 117.34 (C), 110.45 (C), 94.87 (CH), 94.18 (CH), 92.10 (CH), 66.37 (NCH), 56.66 (OCH_3), 56.54 (OCH_3), 56.29 (OCH_3), 31.05 (CH_2), 30.05 (CH_2), 27.00 (CH_2), 26.94 (CH_2), 26.13 (CH_2). **^{19}F NMR (282 MHz, CD_2Cl_2)** δ -73.56 (d, *J* = 710.2 Hz, PF_6). **IR (neat, cm^{-1}):** ν = 3661.34, 2936.30, 2854.21, 2658.44, 2323.09, 2162.63, 2049.70, 1979.82, 1602.09, 1573.73, 1511.57, 1457.79, 1424.79, 1334.22, 1280.35, 1253.82, 1239.66, 1207.50, 1160.63, 1136.14, 1104.54, 1049.91, 1002.30, 947.09, 924.07. **HRMS (ESI)** calculated for [M+] 583.3167, found 583.3150. **m.p.** 202 °C (degradation).

To obtain (*M*) and (*P*) enantiomers, resolution of **2d** was performed with CSP-HPLC (*vide infra*). **OR** (MeCN) (*M*) $[\alpha]_{365}$ +816 and (*P*) $[\alpha]_{365}$ –806.

Chiral Stationary Phase (CSP) HPLC

Enantiomeric excess analysis of **2a**

Columns: CHIRALPAK IB analytic.

Mobile phase:

A: MeOH/EtOH (50:50) + 0.5% Et_3N

B: MeOH/EtOH (50:50) + 0.5% TFA

Elution mixture: A/B = 75:25

Elution: 0.3 mL/min, 10 °C

M-**2a** and *P*-**2a** have been synthetized enantiospecifically starting from enantiopure *M*-**1a** and *P*-**1a** respectively in a previous study.[9]

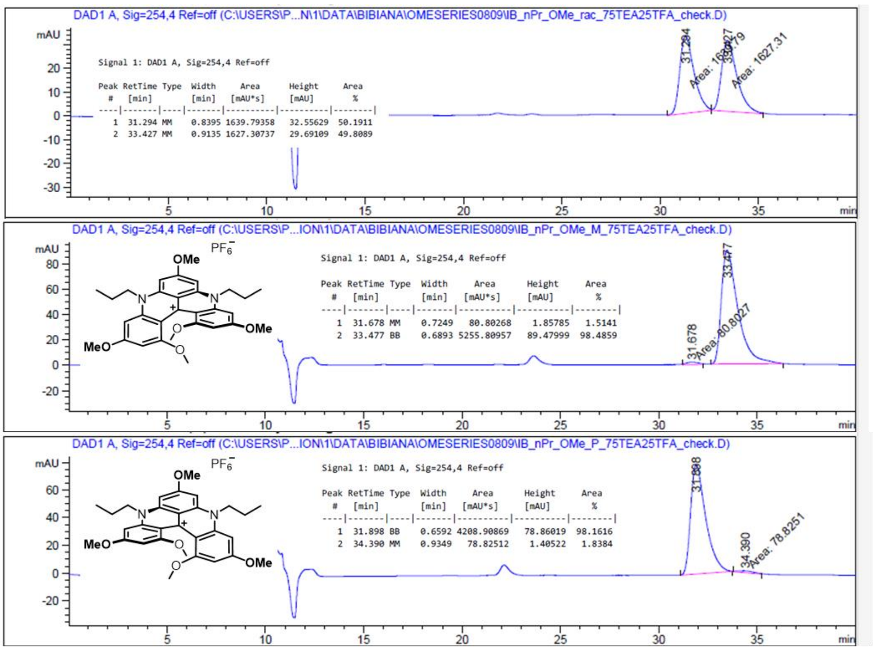

Figure S1. HPLC chromatograms of *rac*-**2a** (first), *M*-**2a** (second) and *P*-**2a** (third).

For derivatives **2b**, **2c** and **2d**, batches of ≈ 10 mg of racemic materials were used leading to 3 to 4 mg of single (*M*) and (*P*) enantiomers.

Resolution of **2b**

Columns: CHIRALPAK IC analytical and semi-preparative.

Mobile phase:

MeOH/EtOH (50:50) + 0.5% Et_3N and 0.3% TFA

Elution: 3.5 mL/min, 15 °C (for semi-preparative) or 1 mL/min, 15 °C (for analytical)

Injection: 50 µL (for semi-preparative) or 30 µL (for analytical)

Concentration: 4 mg/mL

Due to the presence of TFA and Et_3N in the mobile phase, the separated enantiomers solutions were evaporated, dissolved in CH_2Cl_2 and washed with KPF_6 (aq) 0.2 M. Additionally a flash chromatography (SiO_2, CH_2Cl_2/MeOH) was performed and the collected fractions were precipitated with pentane.

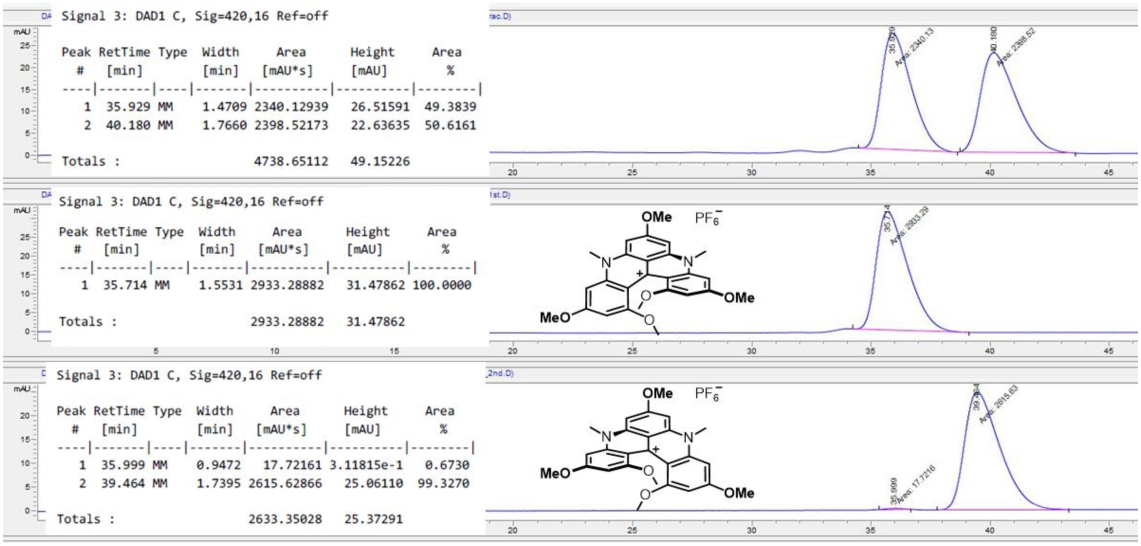

Figure S2. HPLC chromatograms of *rac*-**2b** (first), (*M*)-**2b** (second) and (*P*)-**2b** (third) recorded after HPLC resolution.

According to ECD spectra, and in comparison with the reported unfunctionalized **1b**,[8b] first enantiomer eluted correspond to (*M*)-**2b** and second enantiomer eluted to (*P*)-**2b**.

Resolution of **2c**

Columns: CHIRALPAK IC analytical and semi-preparative.

Mobile phase:

MeOH/EtOH (50:50) + 0.5% Et_3N and 0.3% TFA

Elution: 3.5 mL/min, 15 °C (for semi-preparative) or 1 mL/min, 15 °C (for analytical)

Injection: 40 µL (for semi-preparative) or 30 µL (for analytical)

Concentration: 3.6 mg/mL

Due to the presence of TFA and Et_3N in the mobile phase, the separated enantiomers solutions were evaporated, dissolved in CH_2Cl_2 and washed with KPF_6 (aq) 0.2 M. Additionally a flash chromatography (SiO_2, CH_2Cl_2/MeOH) was performed and the collected fractions were precipitated with pentane.

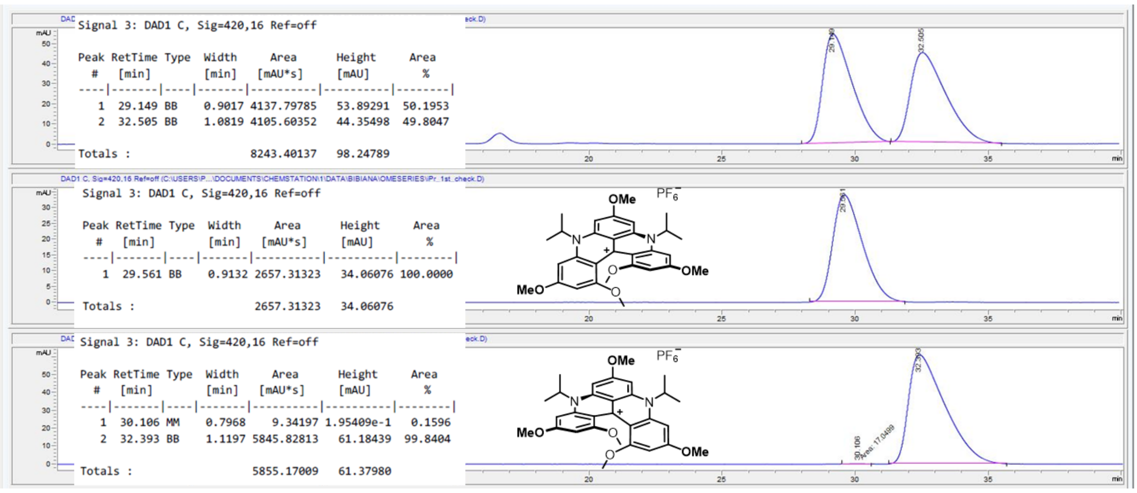

Figure S3. HPLC chromatograms of *rac*-**2c** (first), (*M*)-**2c** (second) and (*P*)- **2c** (third) recorded after HPLC resolution.

According to ECD spectra, and in comparison with the reported unfunctionalized **1c**,[8c] first enantiomer eluted correspond to (*M*)-**2c** and second enantiomer eluted to (*P*)-**2c**.

Resolution of **2d**

Columns: CHIRALPAK IC analytical and semi-preparative.

Mobile phase:

MeOH/EtOH (50:50) + 0.5% Et_3N and 0.3% TFA

Elution: 3.5 mL/min, 15 °C (for semi-preparative) or 1 mL/min, 15 °C (for analytical)

Injection: 50 µL (for semi-preparative) or 20 µL (for analytical)

Concentration: 8.2 mg/mL

Due to the presence of TFA and Et_3N in the mobile phase, the separated enantiomers solutions were evaporated, dissolved in CH_2Cl_2 and washed with KPF_6 (aq) 0.2 M. Additionally a flash chromatography (SiO_2, CH_2Cl_2/MeOH) was performed and the collected fractions were precipitated with pentane.

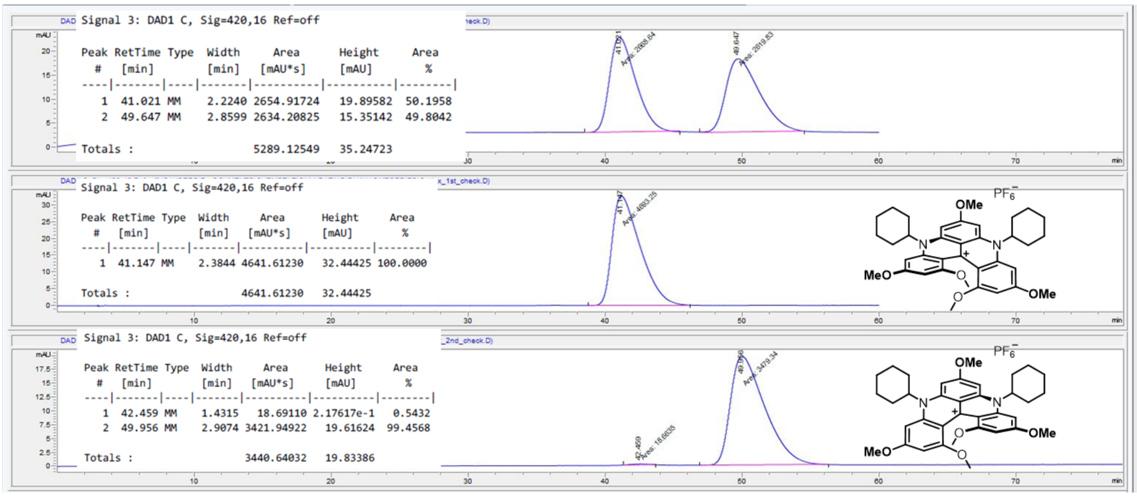

Figure S4. HPLC chromatograms of *rac*-**2d** (first), (*P*)-**2d** (second) and (*M*)-**2d** (third) recorded after HPLC resolution.

According to ECD spectra, and in comparison with the reported unfunctionalized **1d**,[8c] first enantiomer eluted correspond to (*P*)-**2d** and second enantiomer eluted to (*M*)-**2d**.

(Chir)Optical properties

Absorption and emission spectra of compounds **2**

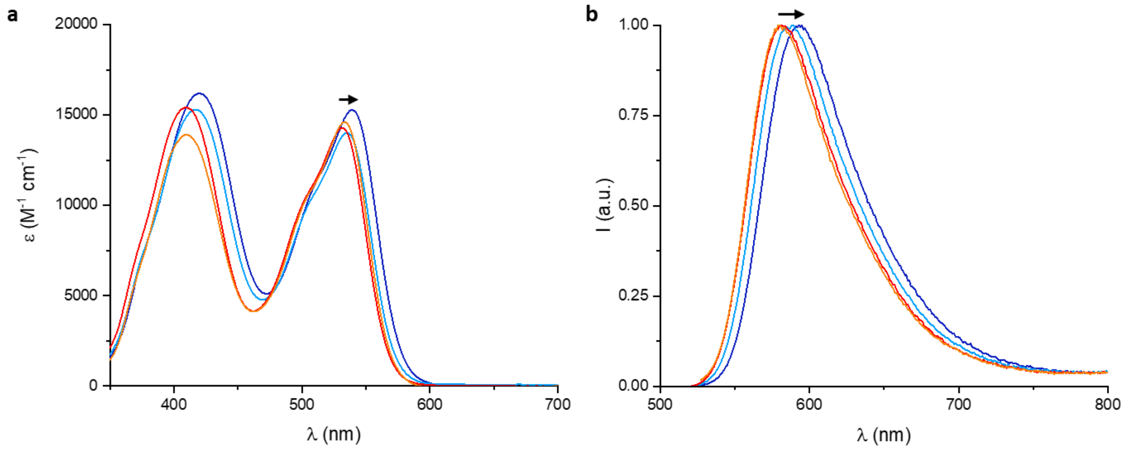

Figure S5. a) Absorption and b) normalized emission spectra of compounds **2a** (orange), **2b** (red), **2c** (light blue) and **2d** (dark blue) in air-equilibrated acetonitrile at RT with concentrations 1×10^{-5} to 5×10^{-6} M.

ECD and CPL spectra of compounds **2a**

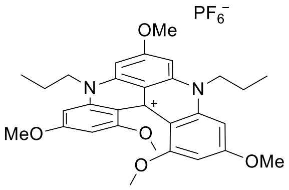

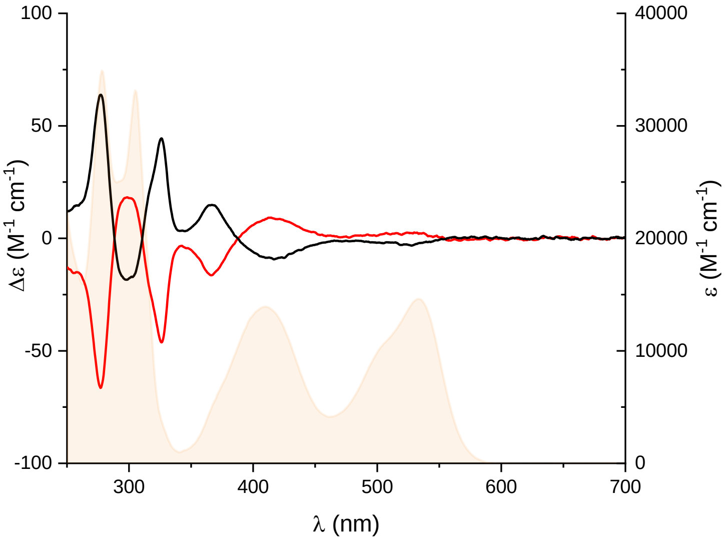

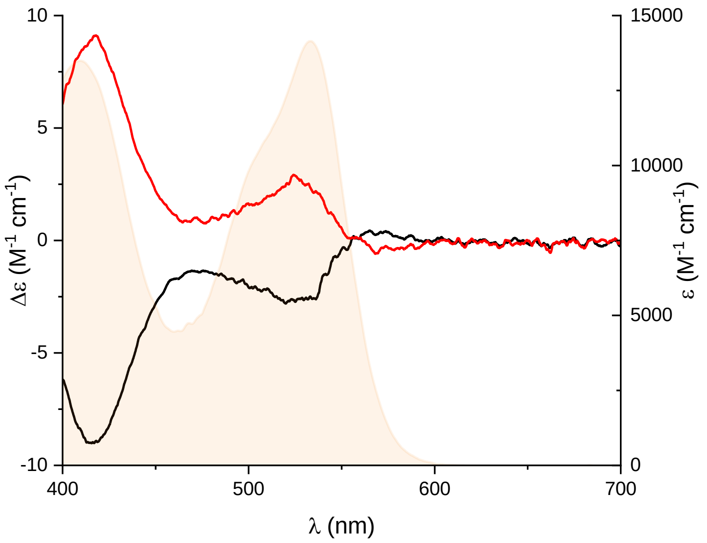

Figure S6. ECD spectra of compound **2a** (*M* enantiomer red and *P* black lines) in air-equilibrated acetonitrile at RT (top) between 250 and 700 nm (C *ca*. 1 ·10^{-5} M) and (bottom) between 400 and 700 nm (C *ca*. 5 ·10^{-5} M). The underlying filled curves correspond to the respective absorption spectrum.

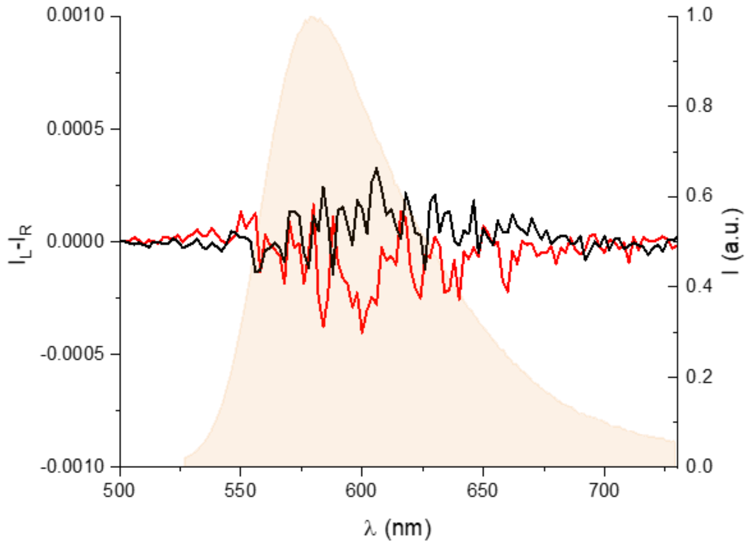

Figure S7. 1PE CPL spectrum of compound **2a** (*M* enantiomer red and *P* black lines) air-equilibrated acetonitrile at RT (C *ca*. 5 ·10^{-6} M). The underlying filled curve corresponds to the respective emission spectrum.

ECD and CPL spectra of compounds **2b**

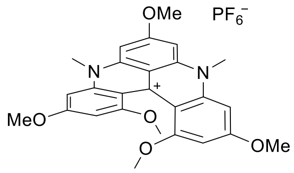

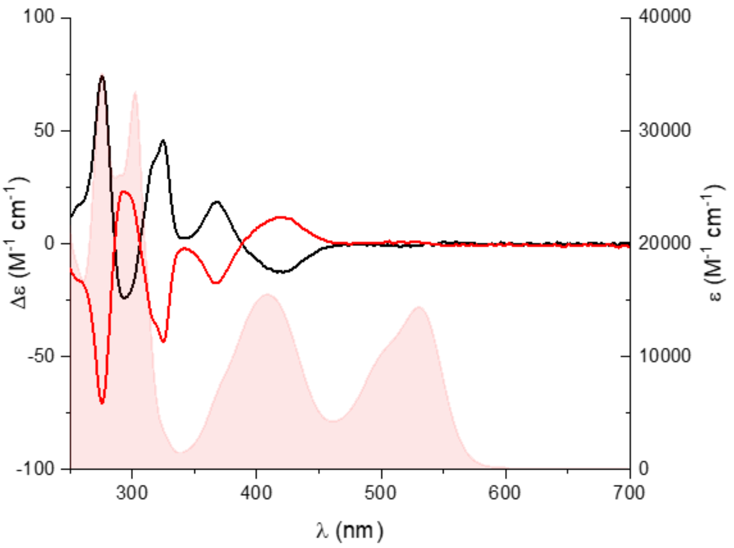

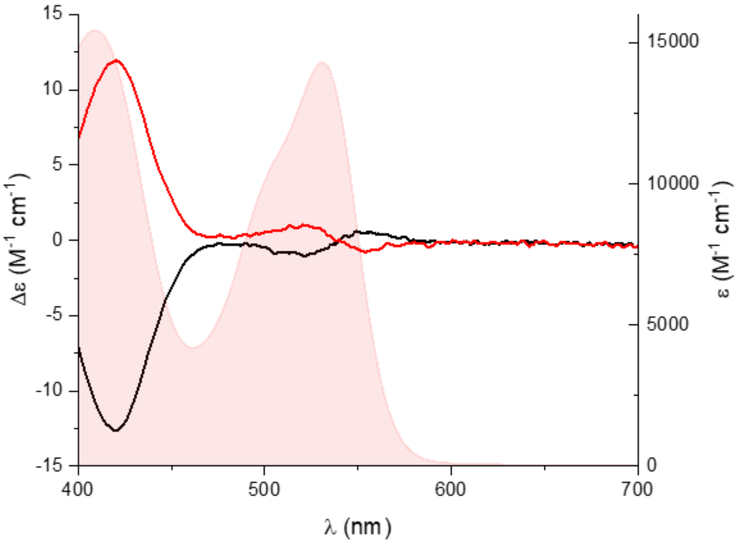

Figure S8. ECD spectra of compound **2b** (*M* enantiomer red and *P* black lines) in air-equilibrated acetonitrile at RT (top) between 250 and 700 nm (C *ca*. 1 $\cdot 10^{-5}$ M) and (bottom) between 400 and 700 nm (C *ca*. 5 $\cdot 10^{-5}$ M). The underlying filled curves correspond to the respective absorption spectrum.

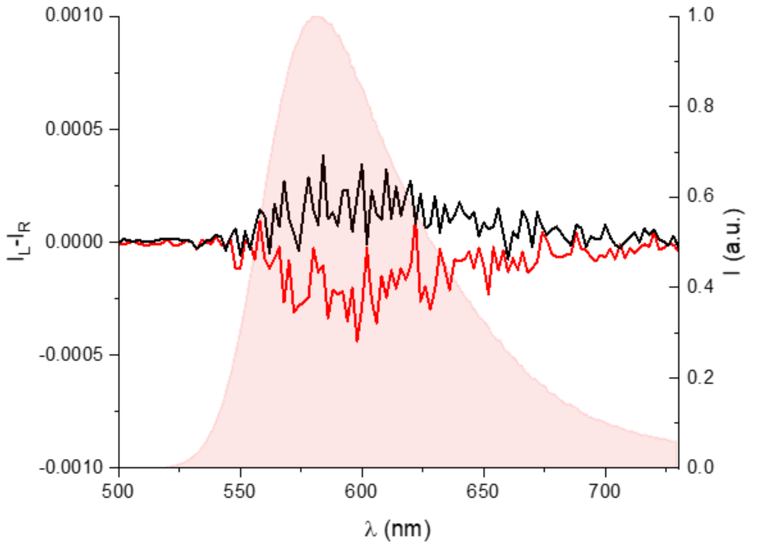

Figure S9. 1PE CPL spectrum of compound **2b** (*M* enantiomer red and *P* black lines) air-equilibrated acetonitrile at RT (C *ca*. 5 ·10^{-6} M). The underlying filled curve corresponds to the respective emission spectrum.

<u>ECD and CPL spectra of compounds **2c**</u>

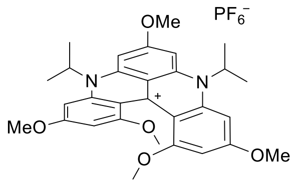

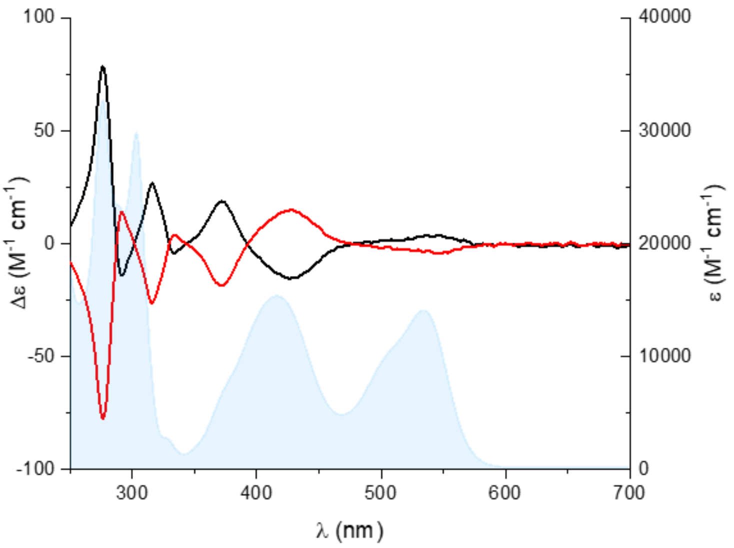

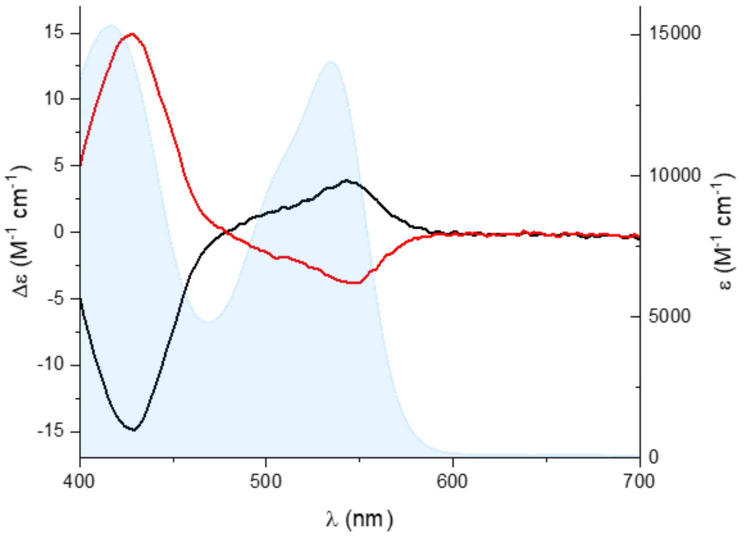

Figure S10. ECD spectra of compound **2c** (*M* enantiomer red and *P* black lines) in air-equilibrated acetonitrile at RT (top) between 250 and 700 nm (C *ca*. 1 $\cdot 10^{-5}$ M) and (bottom) between 400 and 700 nm (C *ca*. 5 $\cdot 10^{-5}$ M). The underlying filled curves correspond to the respective absorption spectrum.

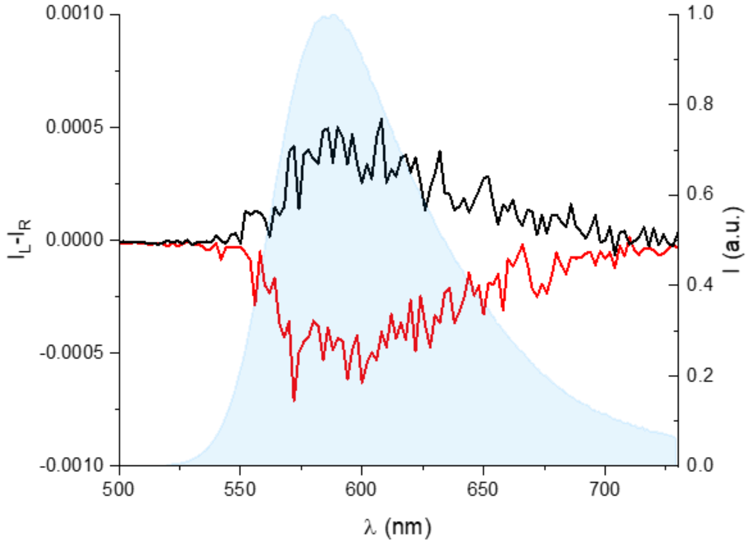

Figure S11. 1PE CPL spectrum of compound **2c** (*M* enantiomer red and *P* black lines) air-equilibrated acetonitrile at RT (C *ca*. 5 $\cdot 10^{-6}$ M). The underlying filled curve corresponds to the respective emission spectrum.

ECD and CPL spectra of compounds **2d**

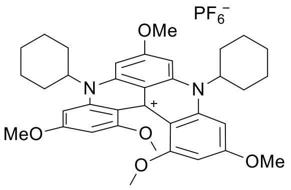

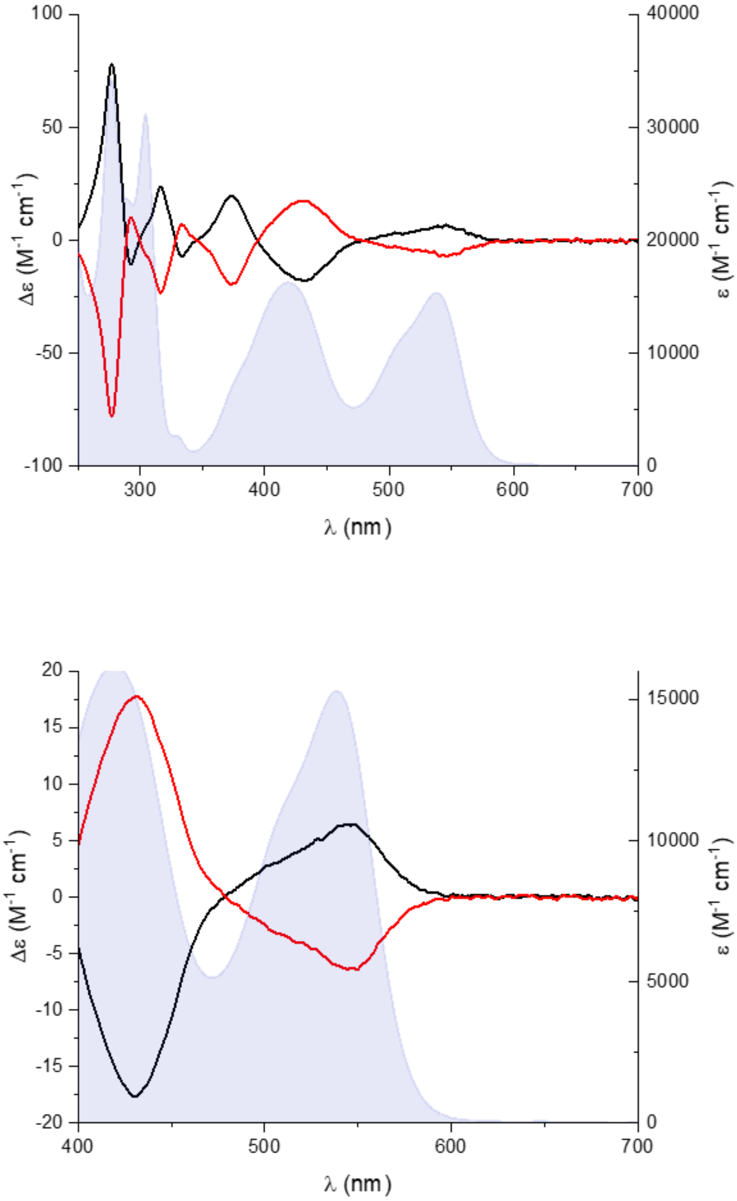

Figure S12. ECD spectra of compound **2d** (*M* enantiomer red and *P* black lines) in air-equilibrated acetonitrile at RT (top) between 250 and 700 nm (C *ca*. 1 $\cdot 10^{-5}$ M) and (bottom) between 400 and 700 nm (C *ca*. 5 $\cdot 10^{-5}$ M). The underlying filled curves correspond to the respective absorption spectrum.

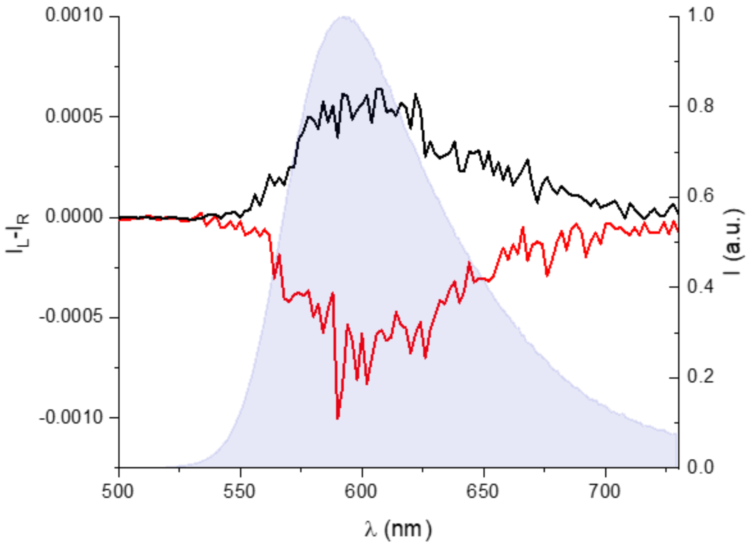

Figure S13. 1PE CPL spectrum of compound **2d** (*M* enantiomer red and *P* black lines) air-equilibrated acetonitrile at RT (C *ca*. 5 ·10^{-6} M). The underlying filled curve corresponds to the respective emission spectrum.

<u>Lifetime measurements</u>

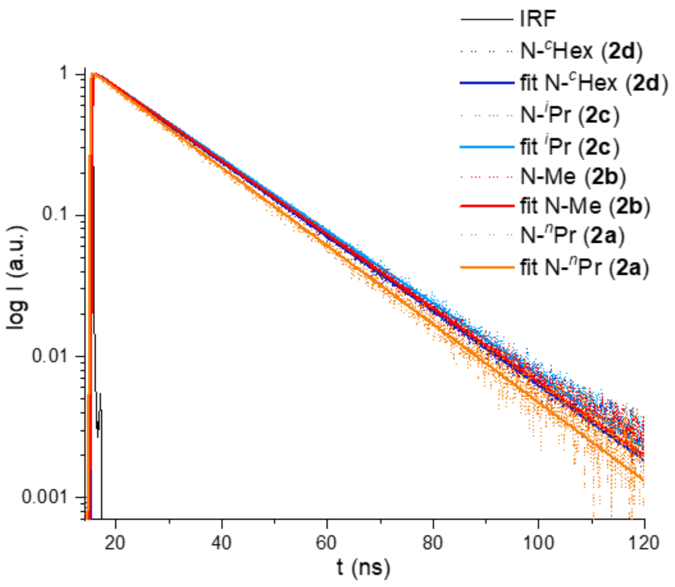

Figure S14. Fluorescence decay of **2a** (orange), **2b** (red), **2c** (light blue) and **2d** (dark blue) in air-equilibrated acetonitrile at RT (C *ca.* 10^{-5} M). The corresponding monoexponential fitting function is shown with a solid line. The instrument response function (IRF) is also reported (black line).

Additional two-photon excitation data

<u>LogI_{em} *vs* LogP_{exc} plot of **2a** with fitting data</u>

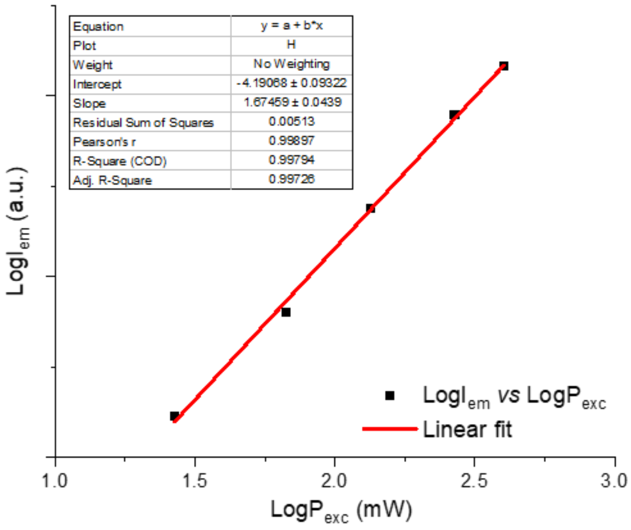

Figure S15. Log *vs* log plot of the excitation-power dependence on the emission maximum (black squares) for compound **2a**. A red line displays the linear fit and all data are shown.

<u>2PE measurements of **2b**</u>

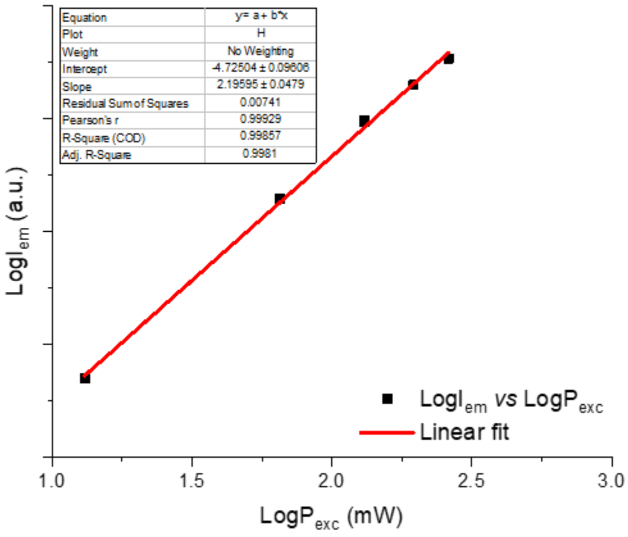

Figure S16. Log *vs* log plot of the excitation-power dependence on the emission maximum (black squares) for compound **2b**. A red line displays the linear fit and all data are shown.

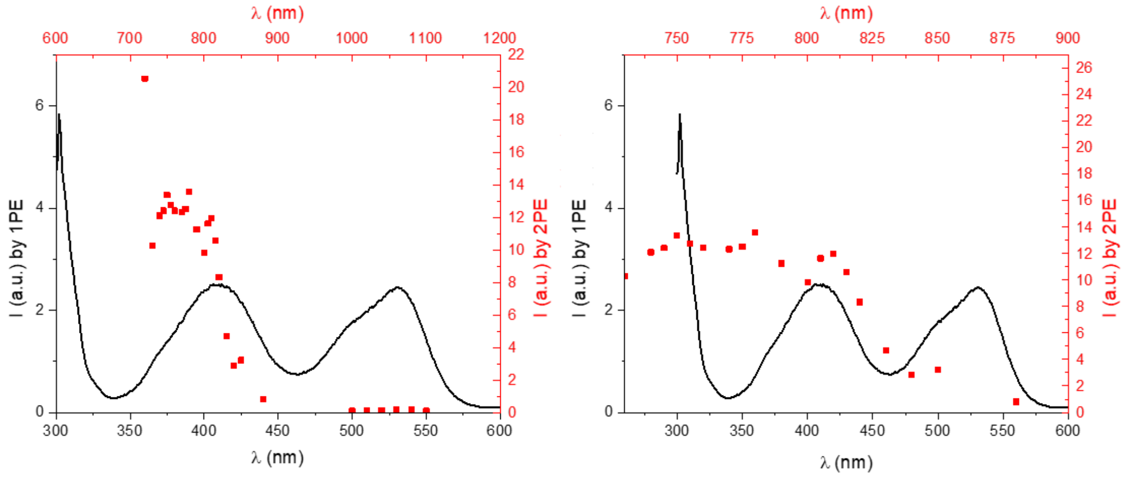

Figure S17. Two-photon excitation (red dots, top x axis) and one-photon excitation (black line, bottom axis) spectra of compound **2b** (λ_{em} = 580 nm) in acetonitrile with concentration around 1 × 10^{-5} M. In the spectra on the right, the x and y axes are adjusted to show better the agreement of the spectral features.

<u>2PE measurements of **2c**</u>

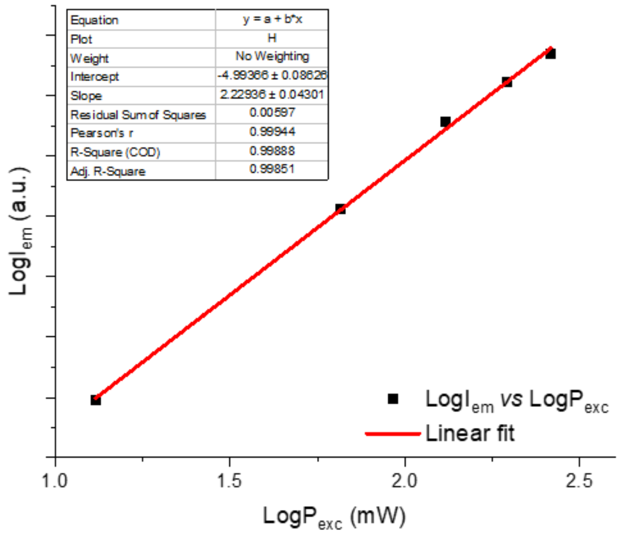

Figure S18. Log *vs* log plot of the excitation-power dependence on the emission maximum (black squares) for compound **2c**. A red line displays the linear fit and all data are shown.

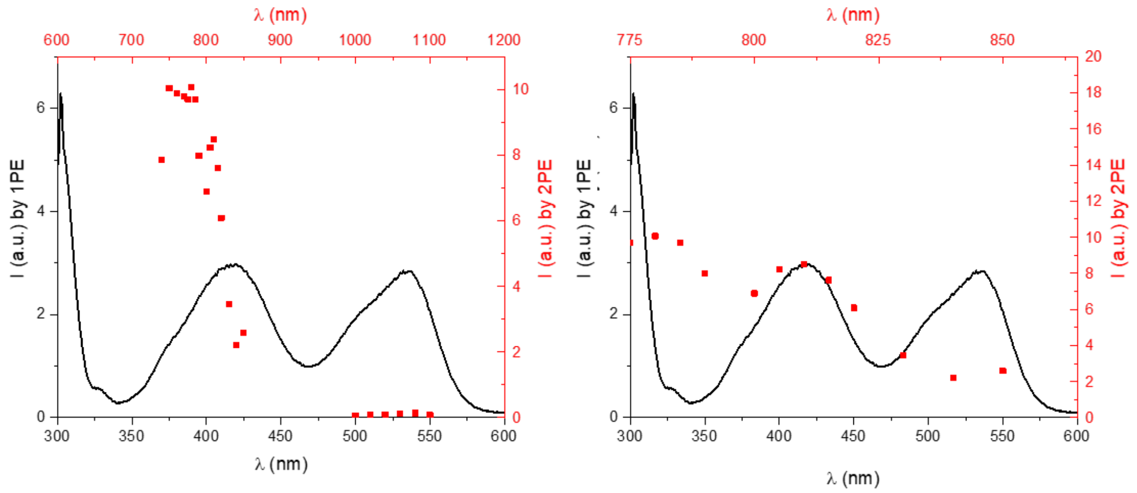

Figure S19. Two-photon excitation (red dots, top x axis) and one-photon excitation (black line, bottom axis) spectra of compound **2c** (λ_{em} = 589 nm) in acetonitrile with concentration around 1 × 10^{-5} M. In the spectra on the right, the x and y axes are adjusted to show better the agreement of the spectral features.

<u>2PE measurements of **2d**</u>

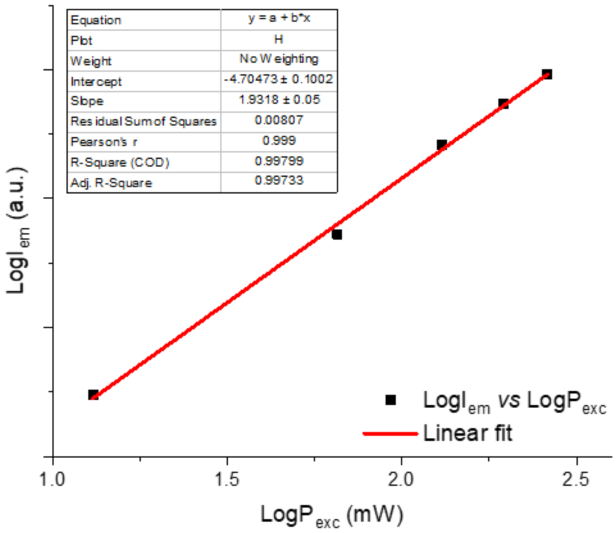

Figure S20. Log *vs* log plot of the excitation-power dependence on the emission maximum (black squares) for compound **2d**. A red line displays the linear fit and all data are shown.

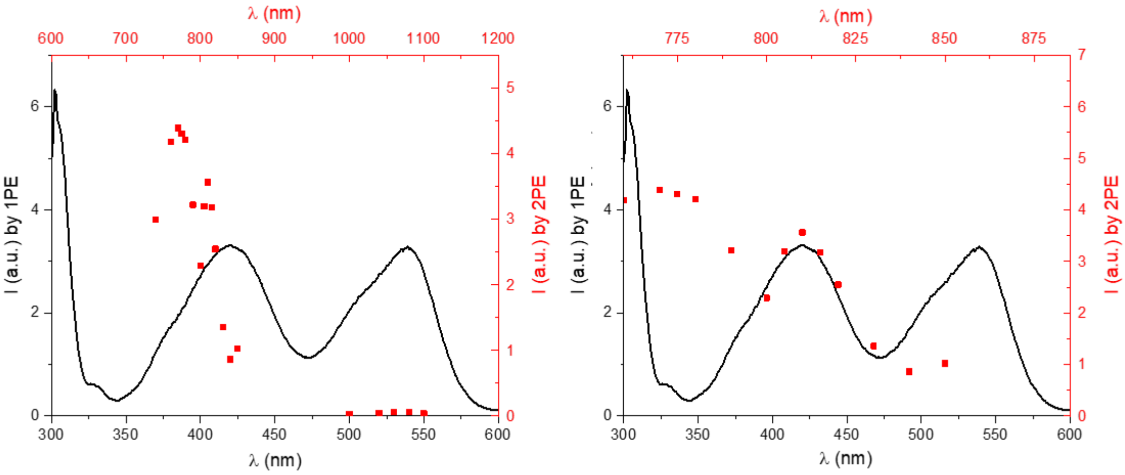

Figure S21. Two-photon excitation (red dots, top x axis) and one-photon excitation (black line, bottom axis) spectra of compound **2d** (λ_{em} = 593 nm) in acetonitrile with concentration around 1 × 10^{-5} M. In the spectra on the right, the x and y axes are adjusted to show better the agreement of the spectral features.

^{1}H, ^{13}C, ^{19}F NMR spectra, IR spectra and HRMS reports of new compounds

Compound **4b**

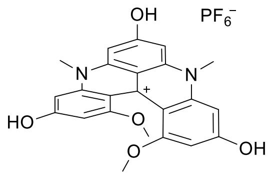

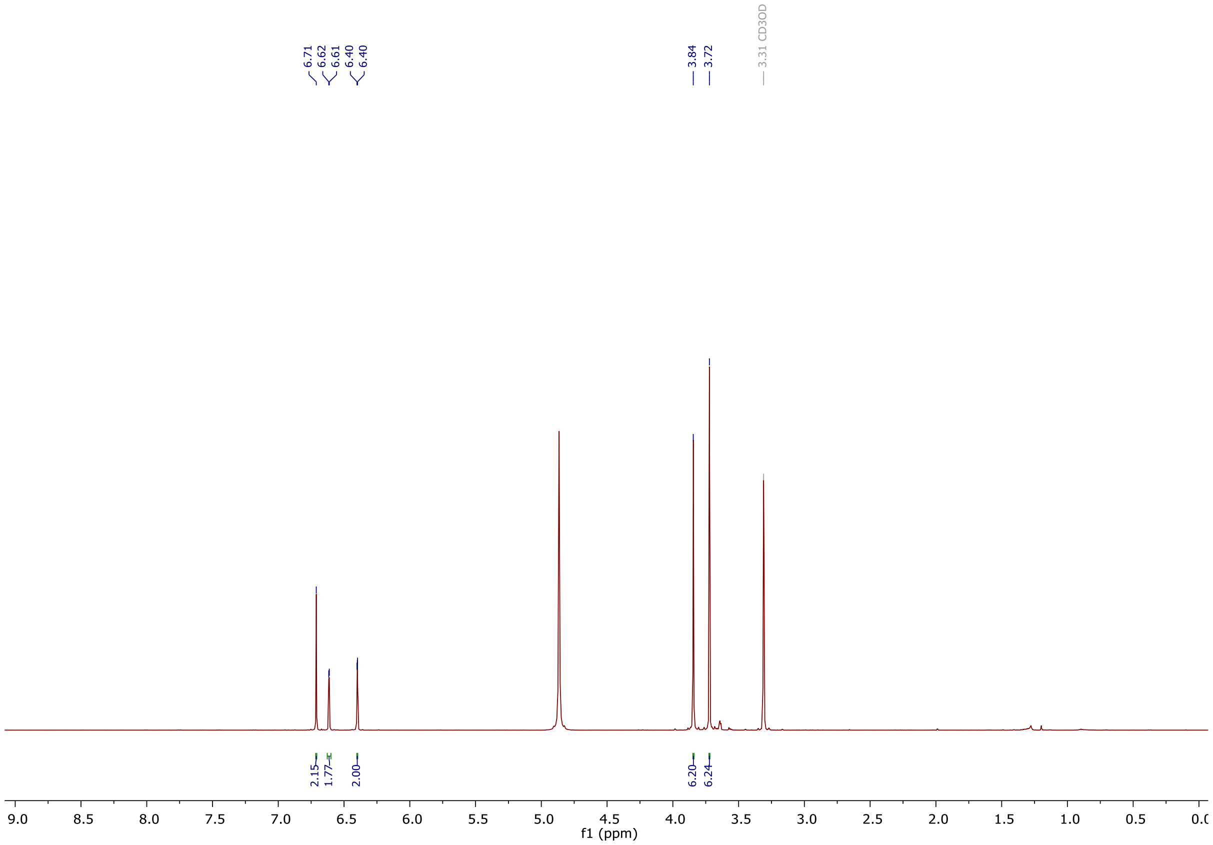

Figure S22. ^{1}H NMR (500 MHz, MeOD) spectrum of **4b**.

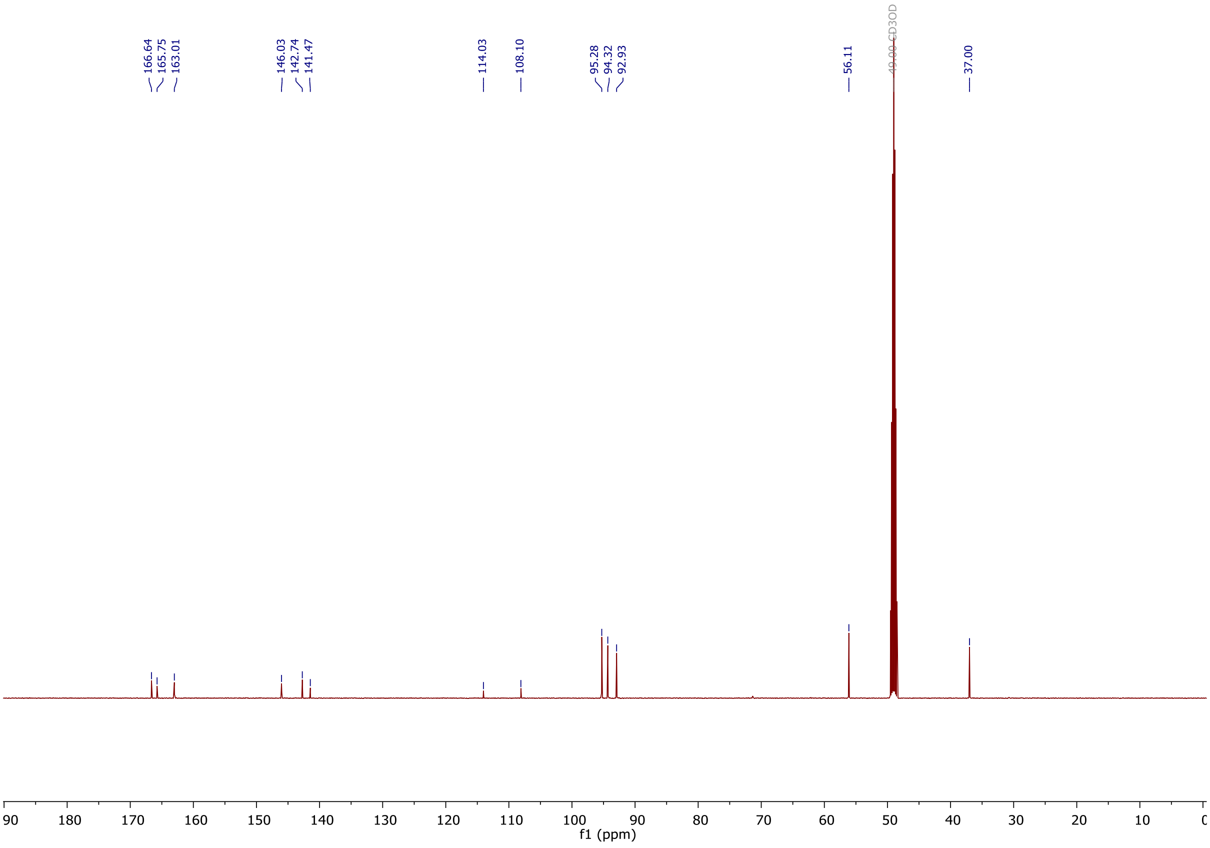

Figure S23. ^{13}C NMR (126 MHz, MeOD) spectrum of **4b**.

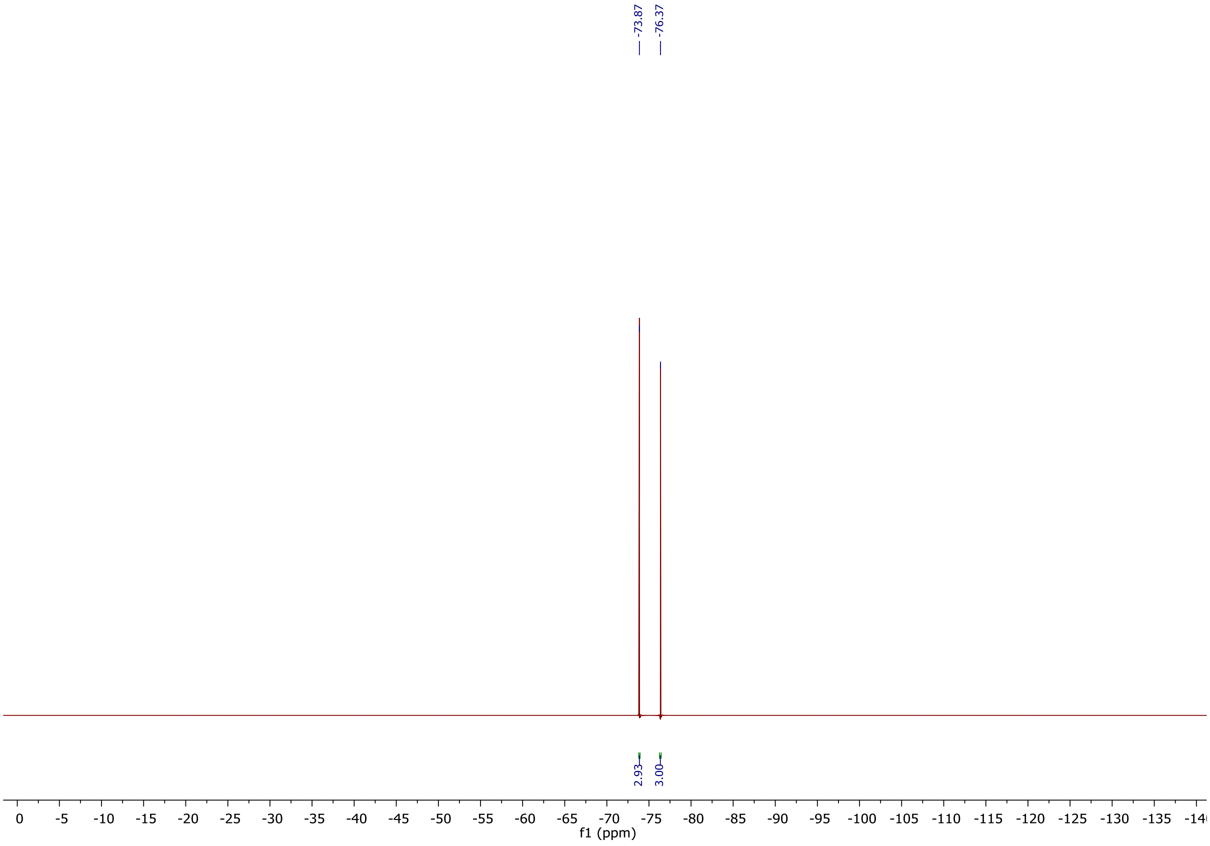

Figure S24. ^{19}F NMR (282 MHz, MeOD) spectrum of **4b**.

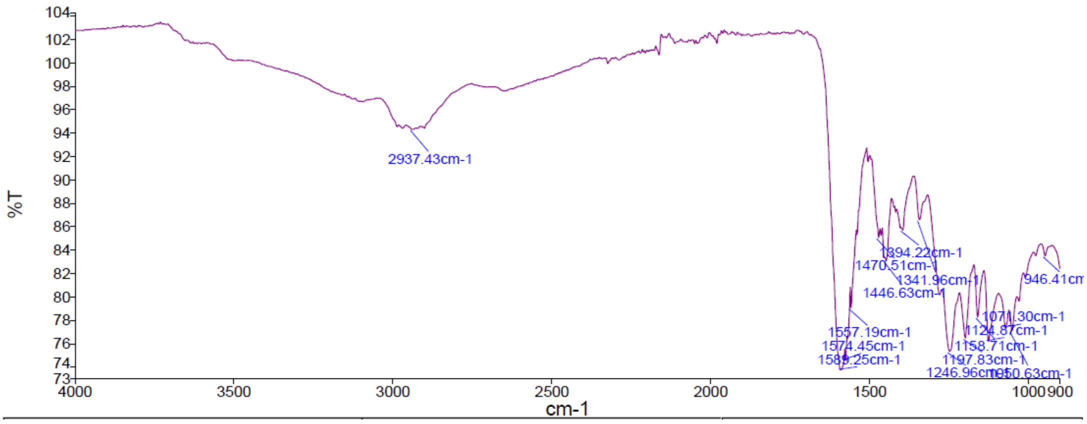

Figure S25. IR (neat) spectrum of **4b**.

Mass Spectrometry Core Facility

Lacour Group – University of Geneva

ESI-HRMS – Certificate of Analysis

Applicant:	Bibiana Fabri	Date of certificate:	14/02/24
Sample name:	BF-253	Instrument:	Xevo G2 Tof (TOF)
Folder:	140224.PRO	Mobile phase:	MeOH (100 µl/min)
Analyst:	Stéphane Grass	Ionisation mode:	ESI (positive polarity)

Elemental Formula	Ion type	Masslynx values *** calc. *m/z*	Masslynx values *** meas. *m/z*	Calc. *m/z*	Meas. *m/z*	Accuracy a) (ppm)
$C_{23}H_{21}N_2O_5$	[M+]	405.1451	405.1454	405.1446	405.1449	0.7

a) *Mass spectrum is calibrated by the use of the MS lockspray system (LeuEnk calibration solution).*

**** MassLynx software does not take into account the mass of the electron for ionic species, therefore the shift of m/z 0.000459.*

Zoomed mass spectrum – Isotopic distribution.

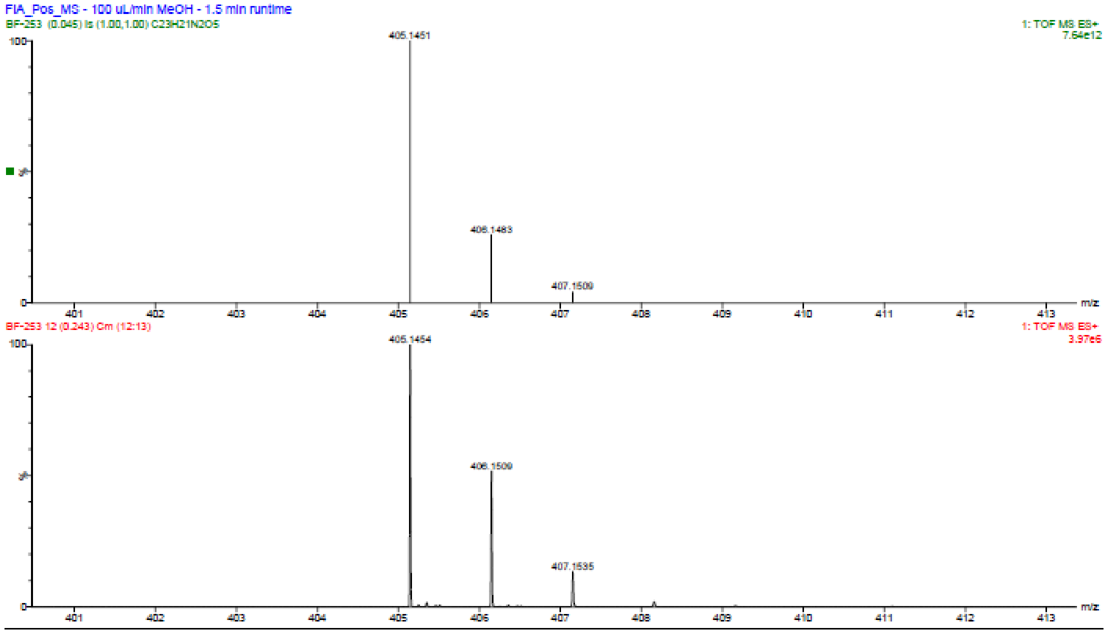

Figure S26. HRMS analysis (ESI, CH_3OH) report of **4b**.

Compound **4c**

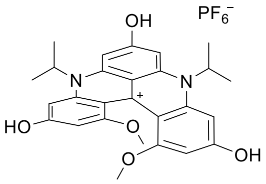

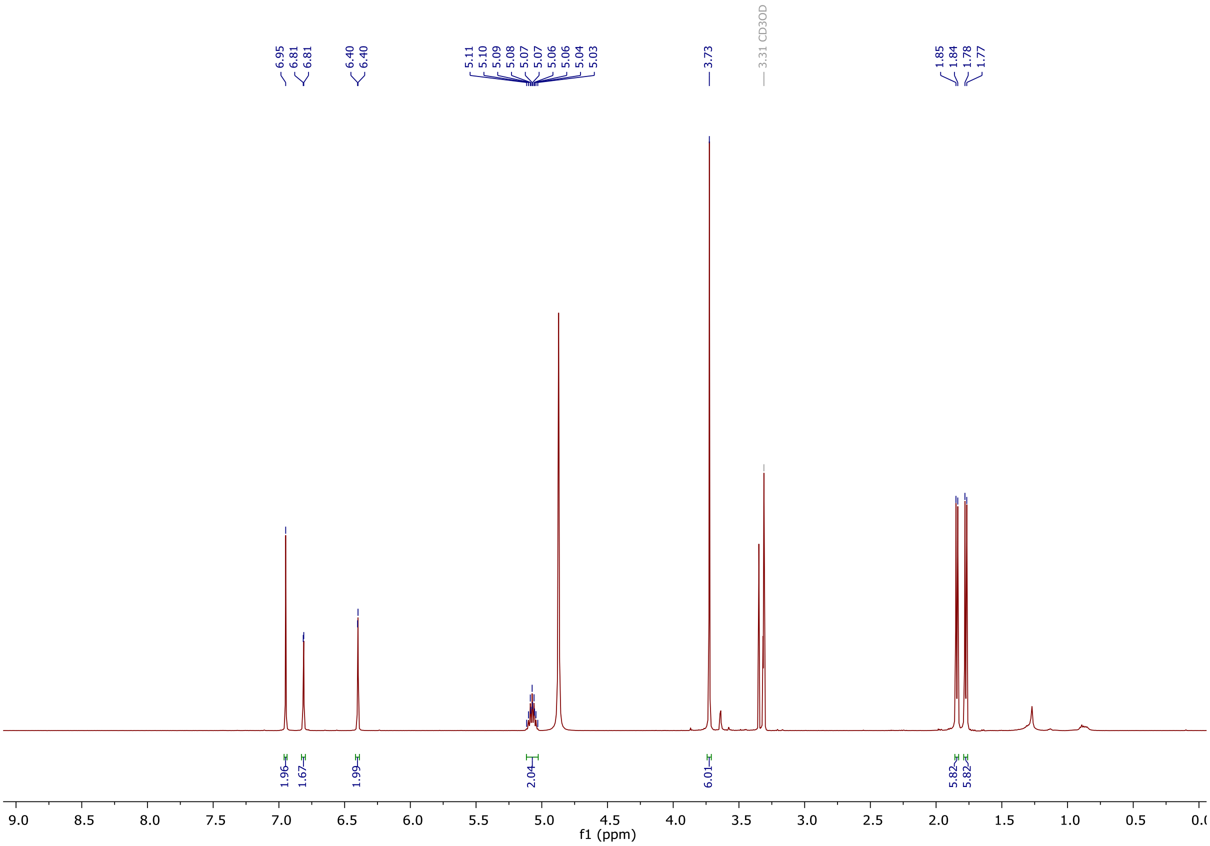

Figure S27. ^{1}H NMR (500 MHz, MeOD) spectrum of **4c**.

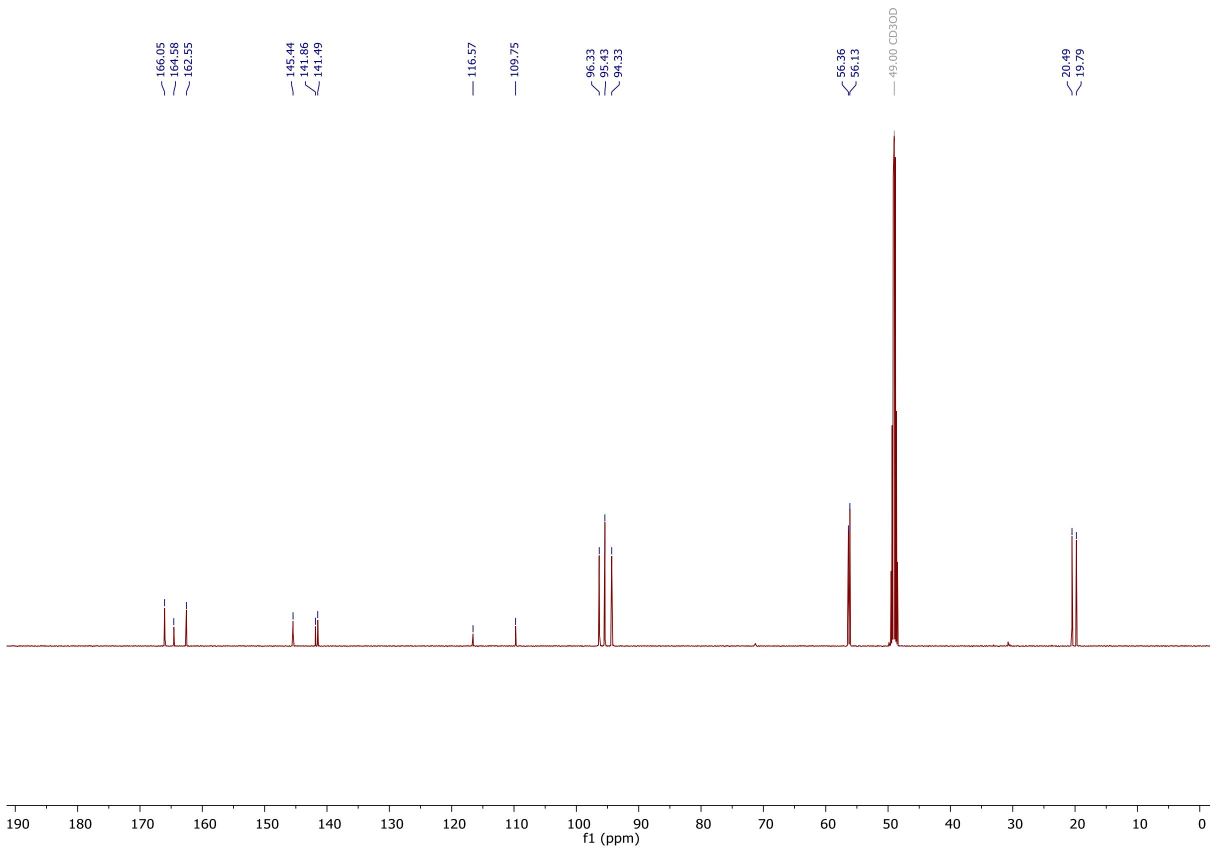

Figure S28. ^{13}C NMR (126 MHz, MeOD) spectrum of **4c**.

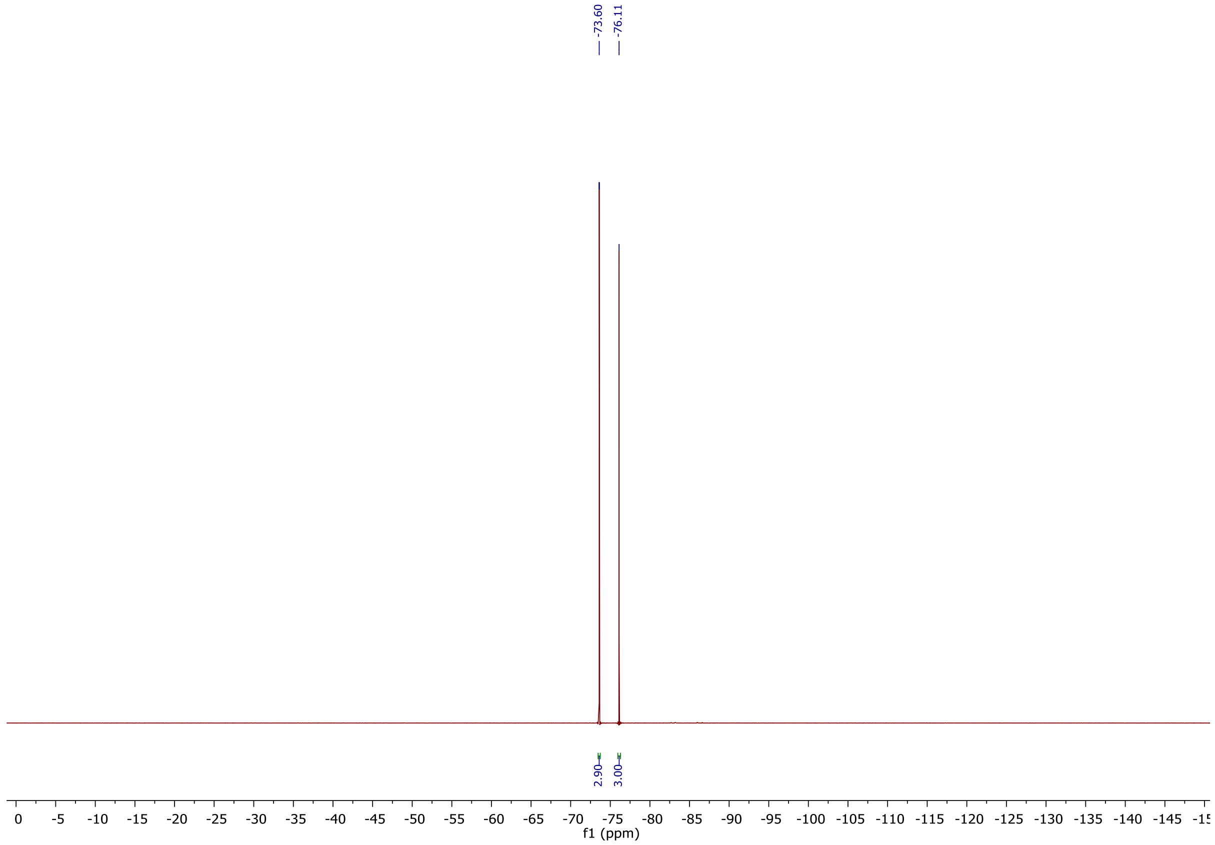

Figure S29. ^{19}F NMR (282 MHz, MeOD) spectrum of **4c**.

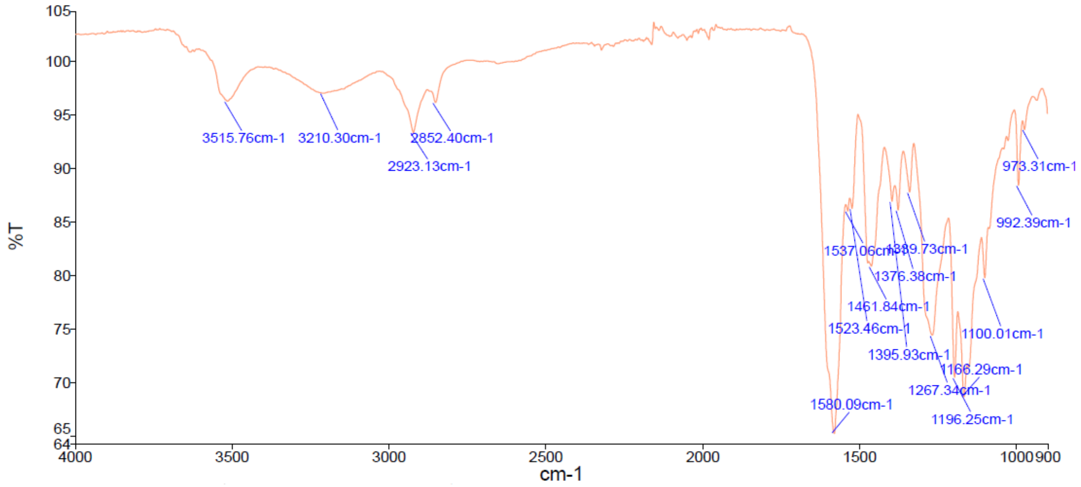

Figure S30. IR (neat) spectrum of **4c**.

Mass Spectrometry Core Facility

Lacour Group – University of Geneva

ESI-HRMS – Certificate of Analysis

Applicant:	Bibiana Fabri	Date of certificate:	14/02/24
Sample name:	BF-250	Instrument:	Xevo G2 Tof (TOF)
Folder:	140224.PRO	Mobile phase:	MeOH (100 µl/min)
Analyst:	Stéphane Grass	Ionisation mode:	ESI (positive polarity)

Elemental Formula	Ion type	Masslynx values *** calc. *m/z*	Masslynx values *** meas. *m/z*	Calc. *m/z*	Meas. *m/z*	Accuracy [a] *(ppm)*
$C_{27}H_{29}N_2O_5$	[M+]	461.2076	461.2085	461.2071	461.2080	2.0

[a] *Mass spectrum is calibrated by the use of the MS lockspray system (LeuEnk calibration solution).*

***** *MassLynx software does not take into account the mass of the electron for ionic species, therefore the shift of m/z 0.000459.*

Zoomed mass spectrum – Isotopic distribution.

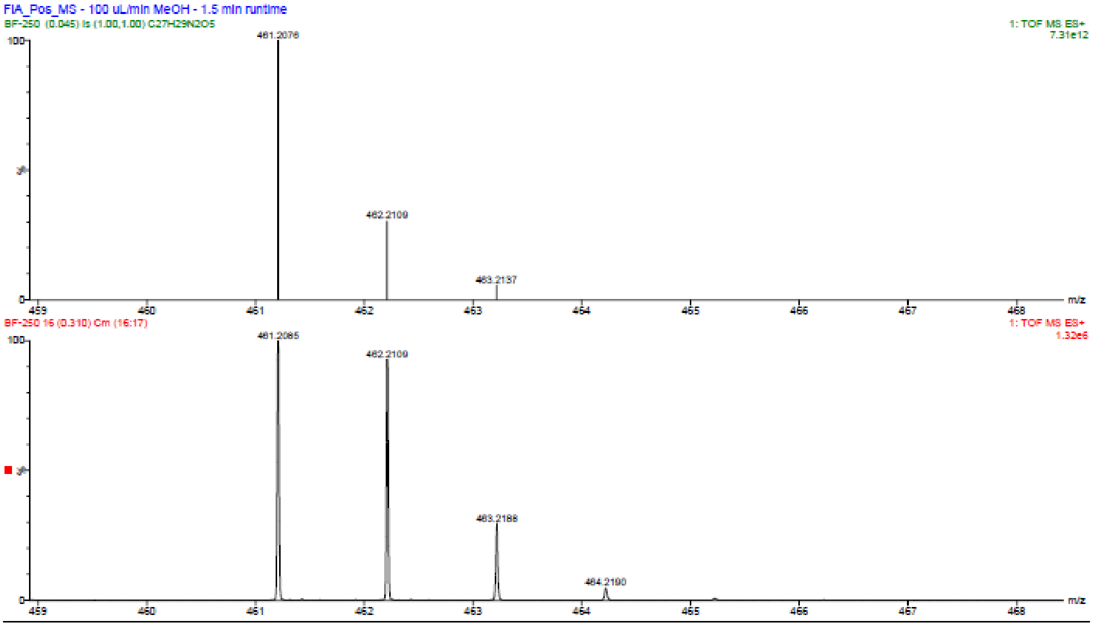

Figure S31. HRMS analysis (ESI, CH_3OH) report of **4c**.

Compound **4d**

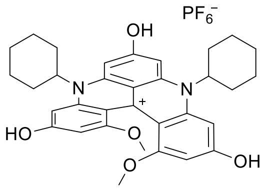

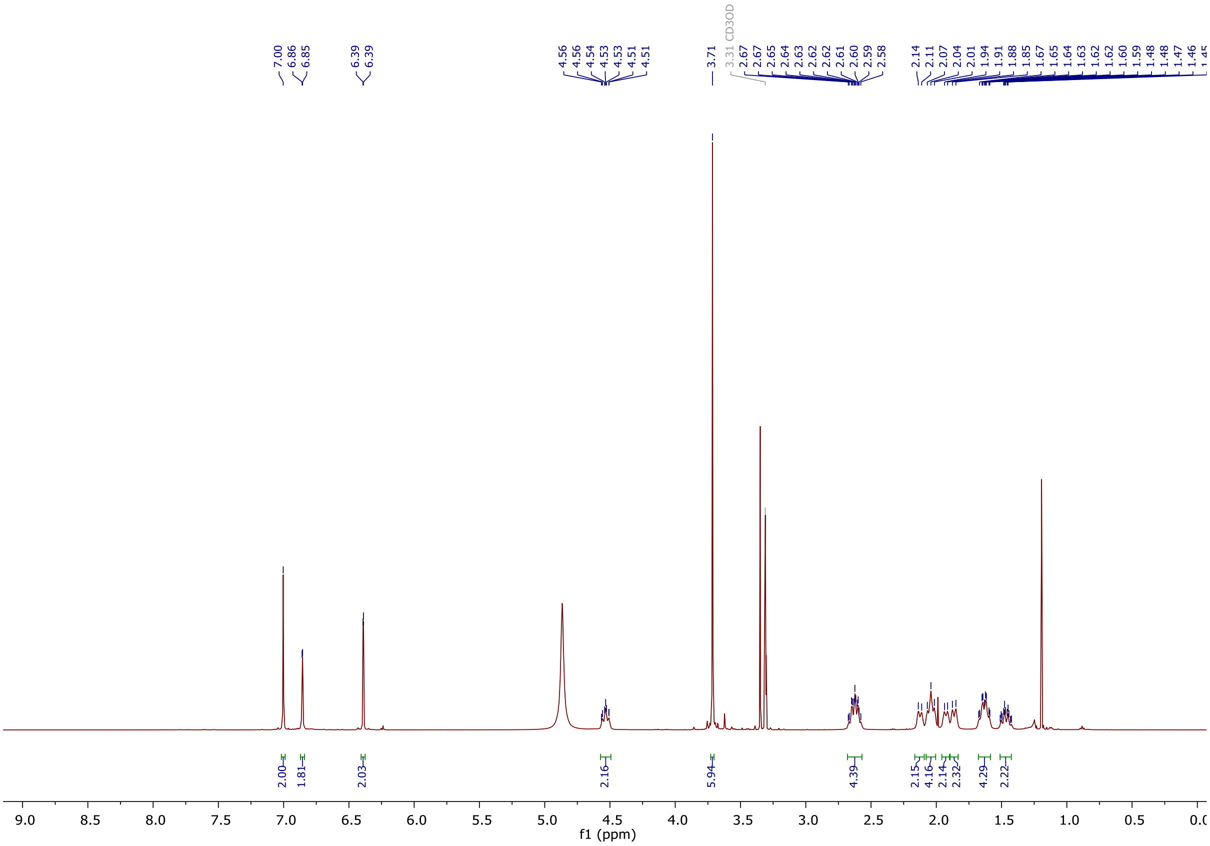

Figure S32. ^{1}H NMR (500 MHz, MeOD) spectrum of **4d**.

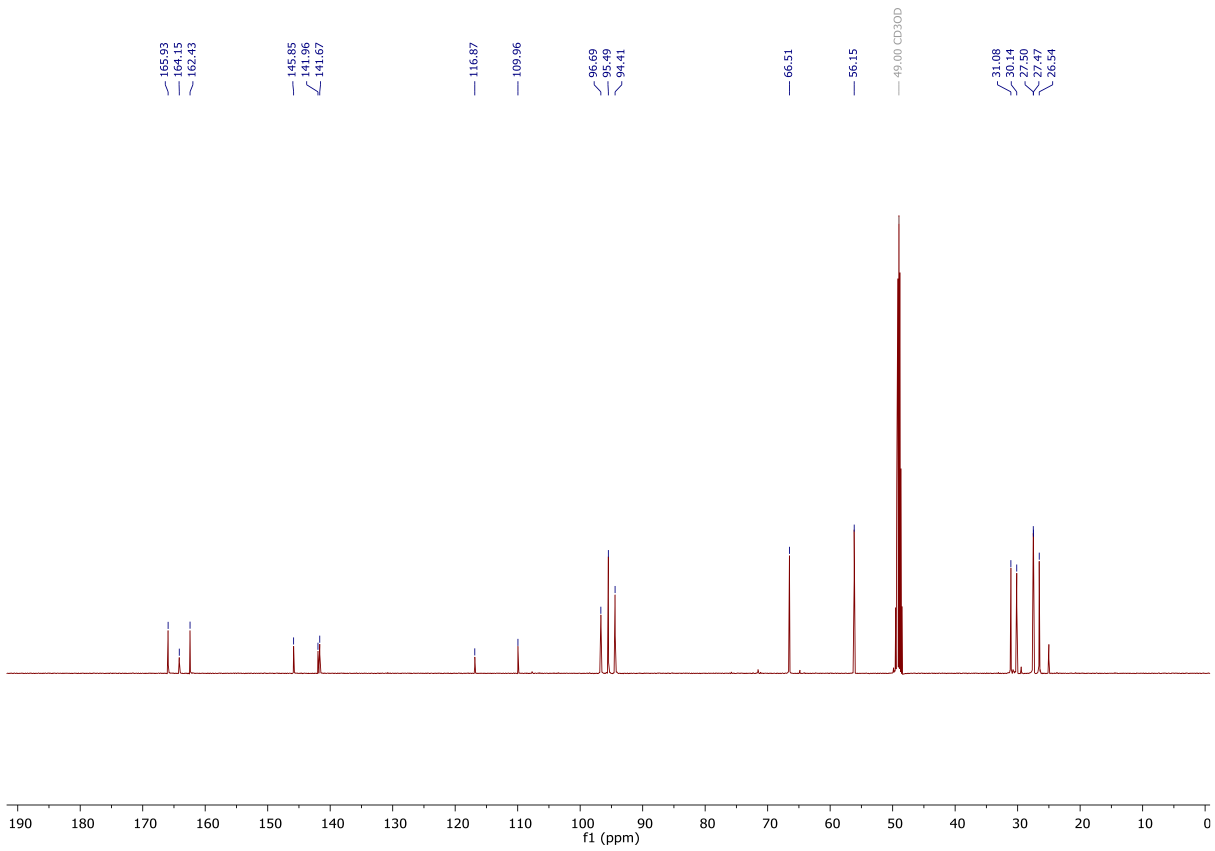

Figure S33. ^{13}C NMR (126 MHz, MeOD) spectrum of **4d**.

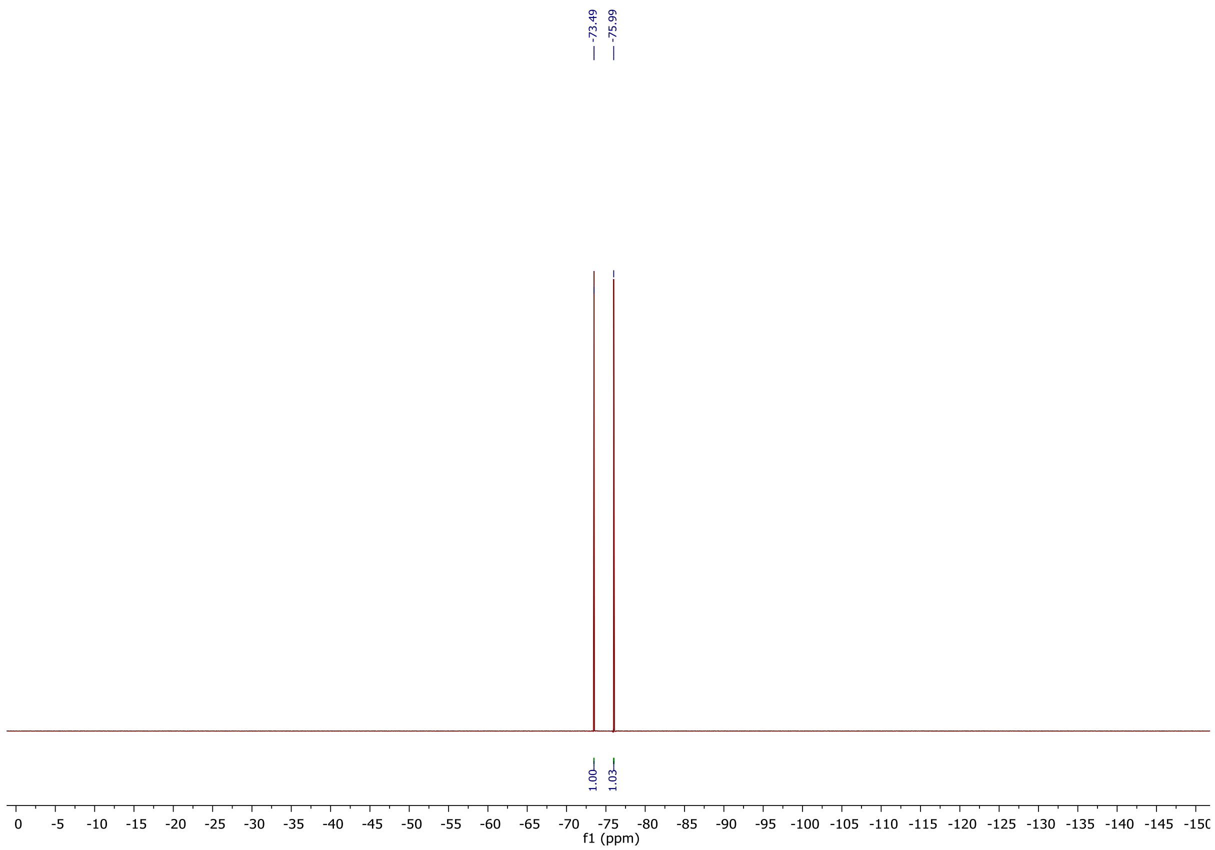

Figure S34. ^{19}F NMR (282 MHz, MeOD) spectrum of **4d**.

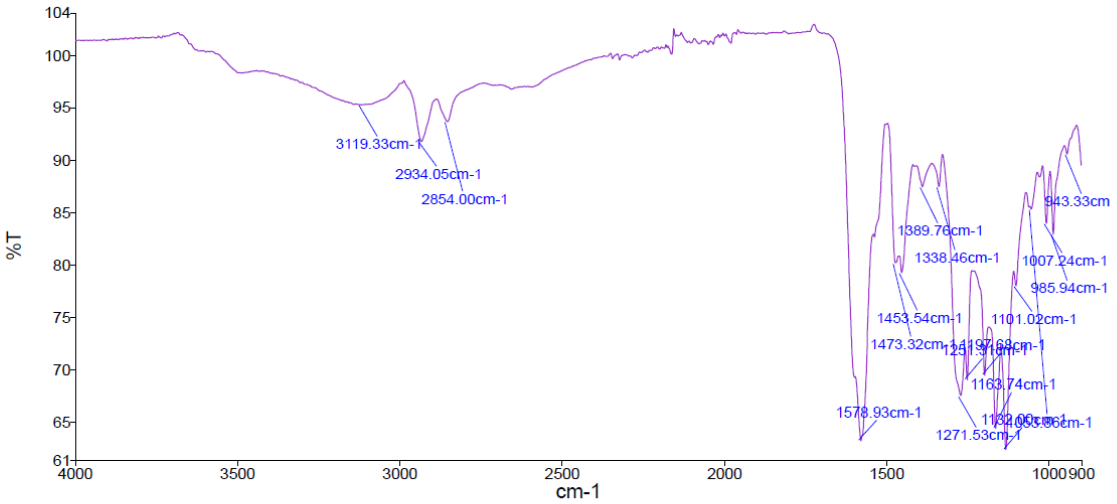

Figure S35. IR (neat) spectrum of **4d**.

Mass Spectrometry Core Facility

Lacour Group – University of Geneva

ESI-HRMS – Certificate of Analysis

Applicant:	Bibiana Fabri	Date of certificate:	14/02/24
Sample name:	BF-255	Instrument:	Xevo G2 Tof (TOF)
Folder:	140224.PRO	Mobile phase:	MeOH (100 µl/min)
Analyst:	Stéphane Grass	Ionisation mode:	ESI (positive polarity)

Elemental Formula	Ion type	*Masslynx values **** calc. m/z	meas. m/z	Calc. *m/z*	Meas. *m/z*	Accuracy [a] (ppm)
$C_{33}H_{37}N_2O_5$	[M+]	541.2703	541.2723	541.2698	541.2718	3.7

[a] *Mass spectrum is calibrated by the use of the MS lockspray system (LeuEnk calibration solution).*

**** MassLynx software does not take into account the mass of the electron for ionic species, therefore the shift of m/z 0.000459.*

Zoomed mass spectrum – Isotopic distribution.

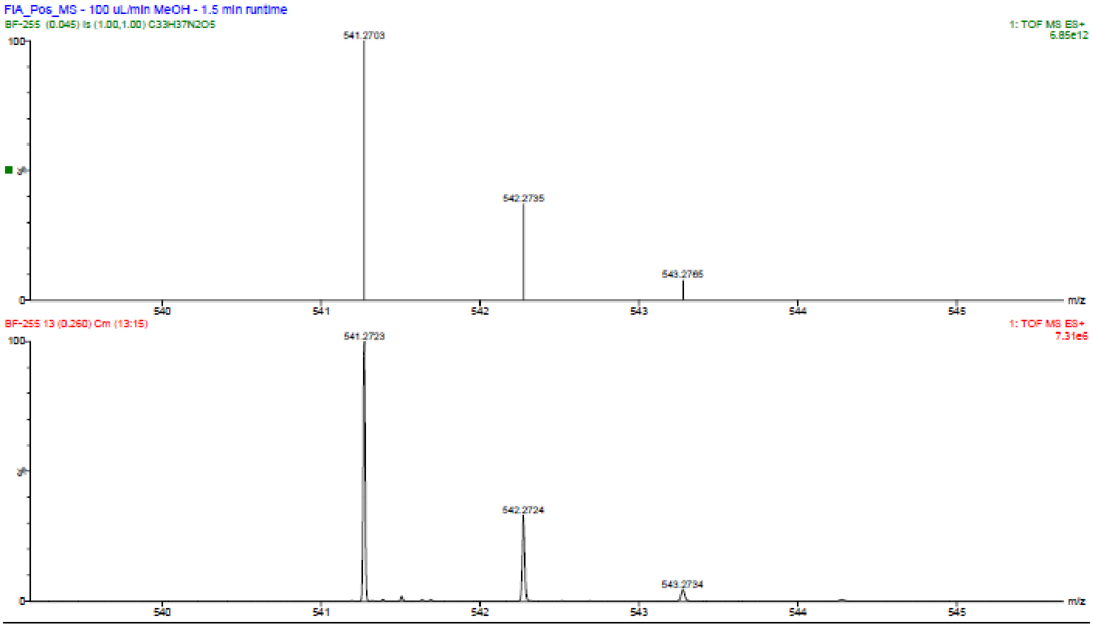

Figure S36. HRMS analysis (ESI, CH_3OH) report of **4d**.

Compound **2b**

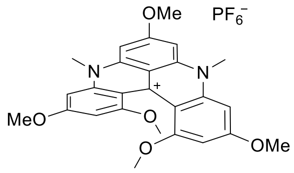

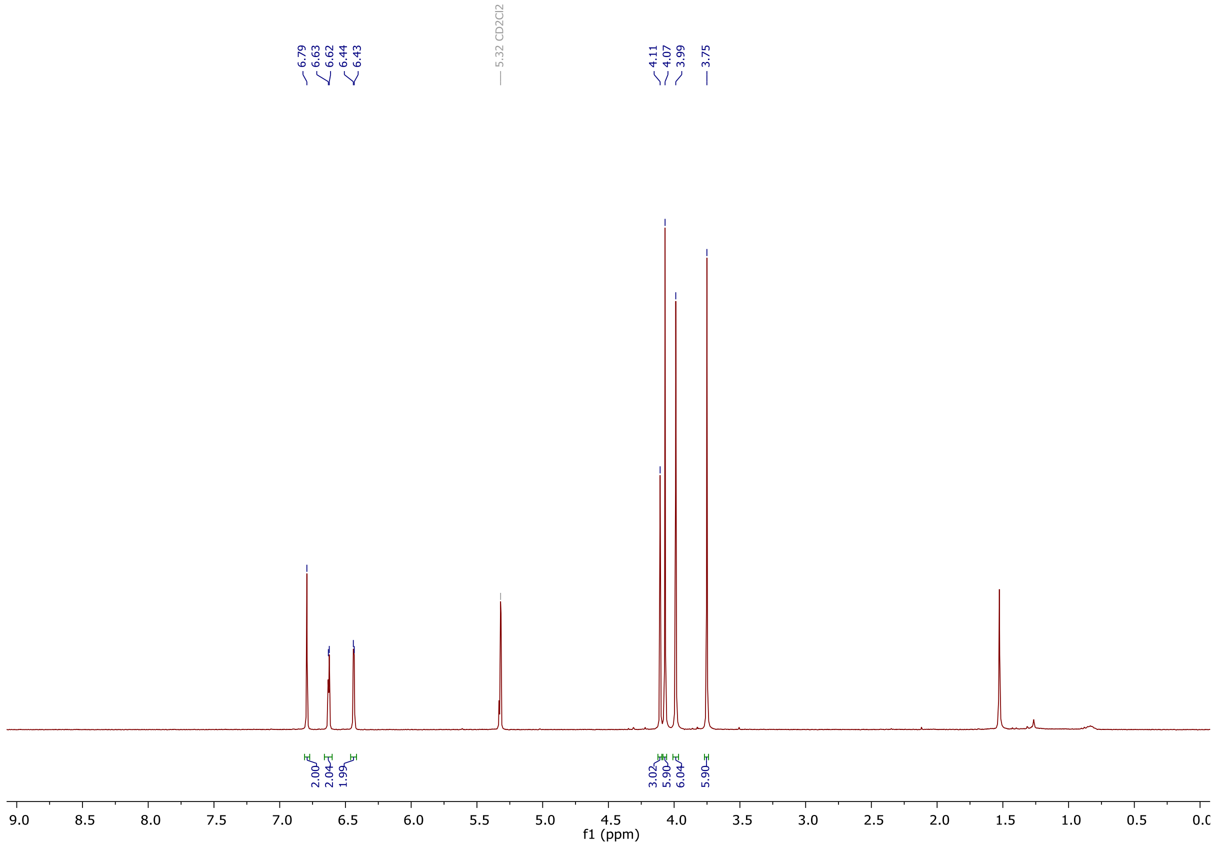

Figure S37. ^{1}H NMR (500 MHz, CD_2Cl_2) spectrum of **2b**.

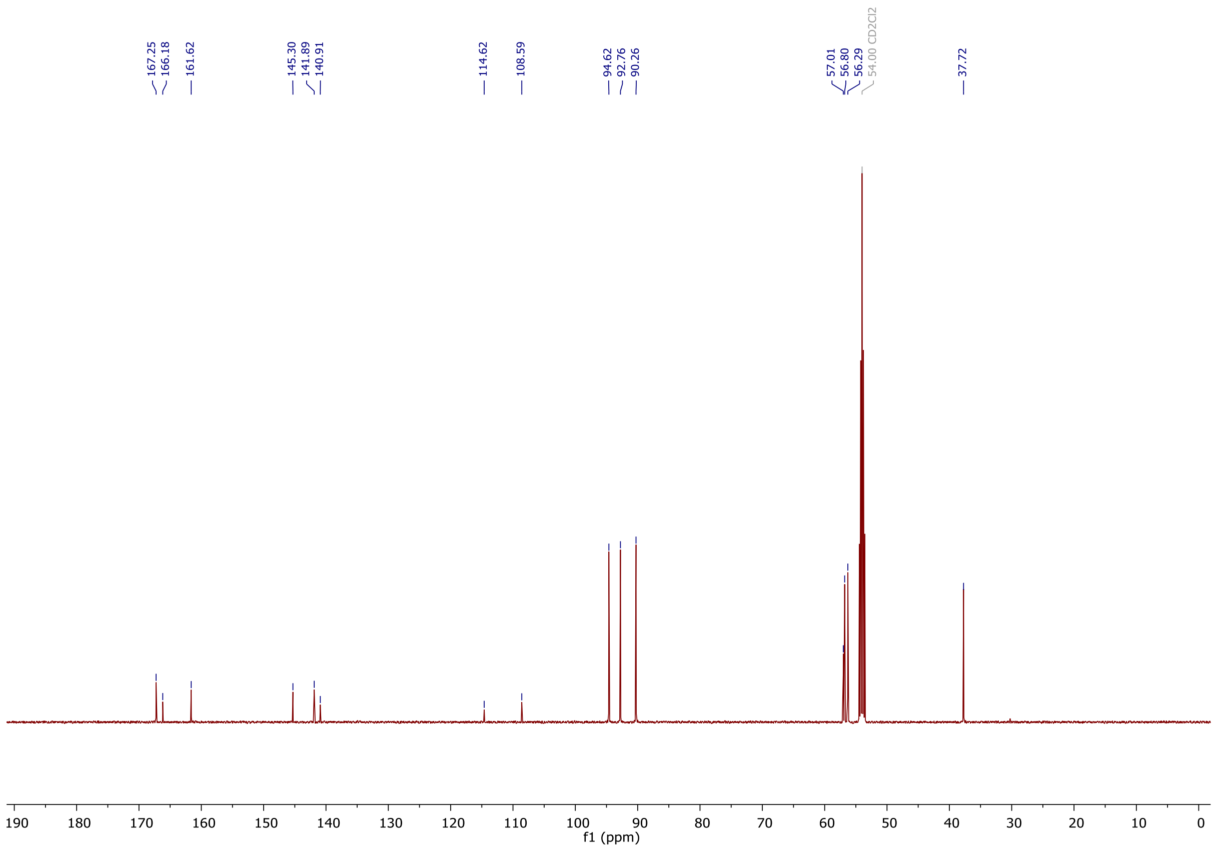

Figure S38. ^{13}C NMR (126 MHz, CD_2Cl_2) spectrum of **2b**.

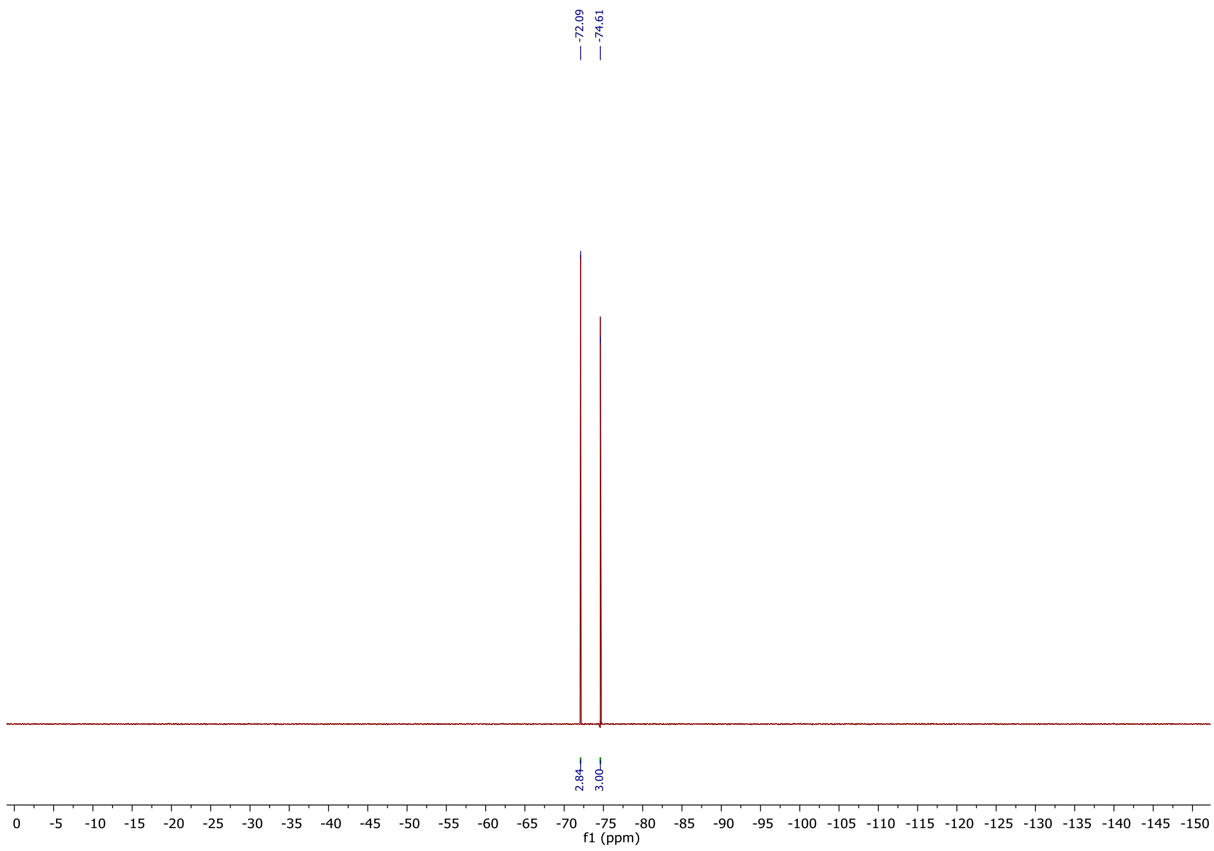

Figure S39. ^{19}F NMR (282 MHz, CD_2Cl_2) spectrum of **2b**.

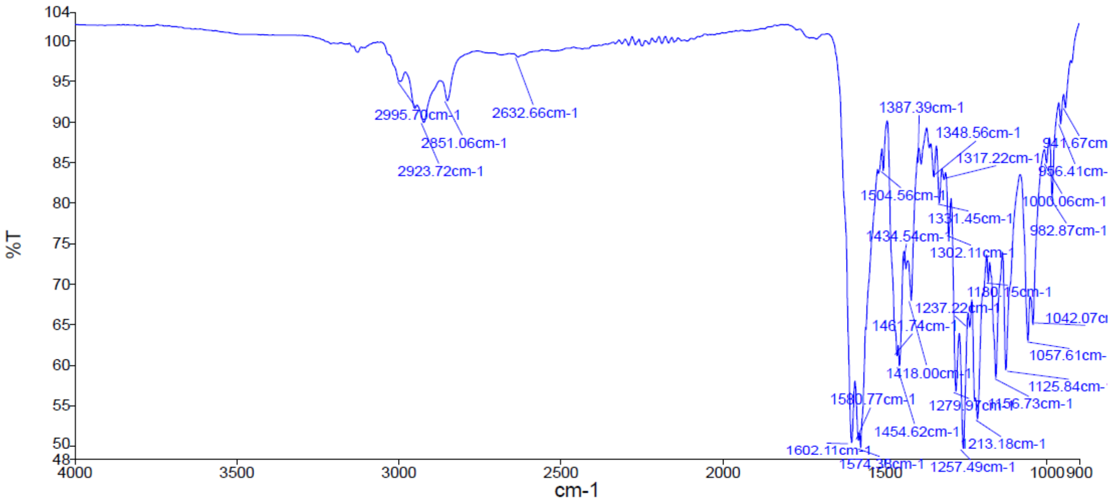

Figure S40. IR (neat) spectrum of **2b**.

Mass Spectrometry Core Facility

Lacour Group – University of Geneva

ESI-HRMS – Certificate of Analysis

Applicant:	Bibiana Fabri	Date of certificate:	03/10/23
Sample name:	BF-NMe	Instrument:	Xevo G2 Tof (TOF)
Folder:	031023.PRO	Mobile phase:	MeOH (100 µl/min)
Analyst:	Stéphane Grass	Ionisation mode:	ESI (positive polarity)

Elemental Formula	Ion type	Masslynx values *** calc. m/z	meas. m/z	Calc. *m/z*	Meas. *m/z*	Accuracy [a] (ppm)
$C_{26}H_{27}N_2O_5$	[M+]	447.192	447.1940	447.1915	447.1935	4.5

[a] *Mass spectrum is calibrated by the use of the MS lockspray system (LeuEnk calibration solution).*

**** MassLynx software does not take into account the mass of the electron for ionic species, therefore the shift of m/z 0.000459.*

Zoomed mass spectrum – Isotopic distribution.

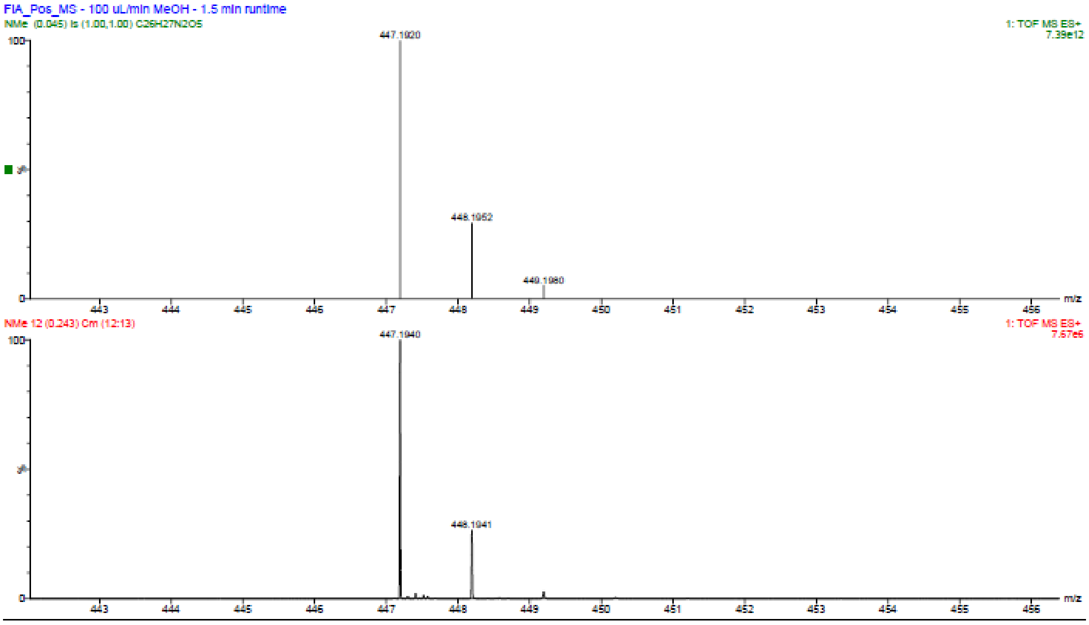

Figure S41. HRMS analysis (ESI, CH_3OH) report of **2b**.

Compound **2c**

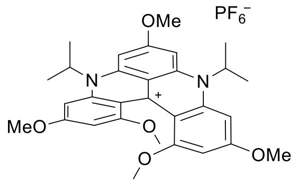

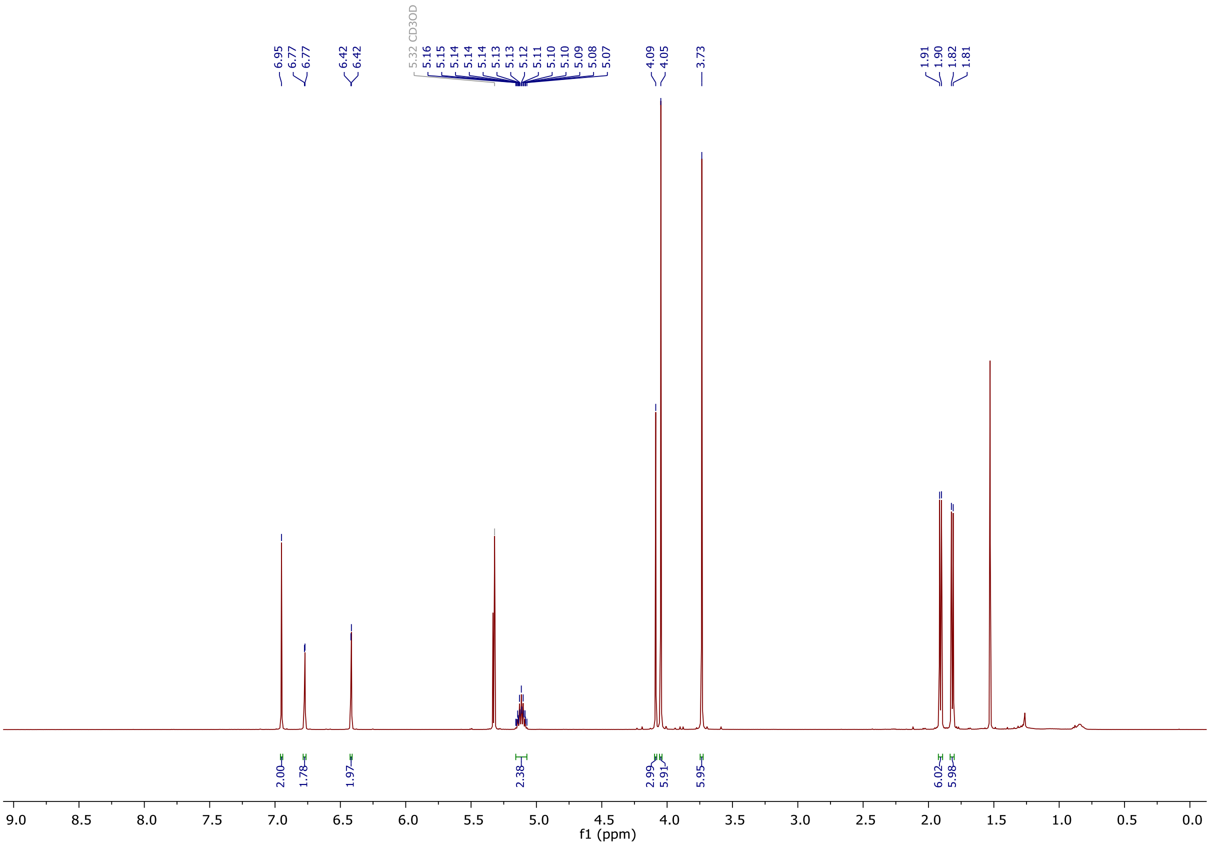

Figure S42. ^{1}H NMR (500 MHz, CD_2Cl_2) spectrum of **2c**.

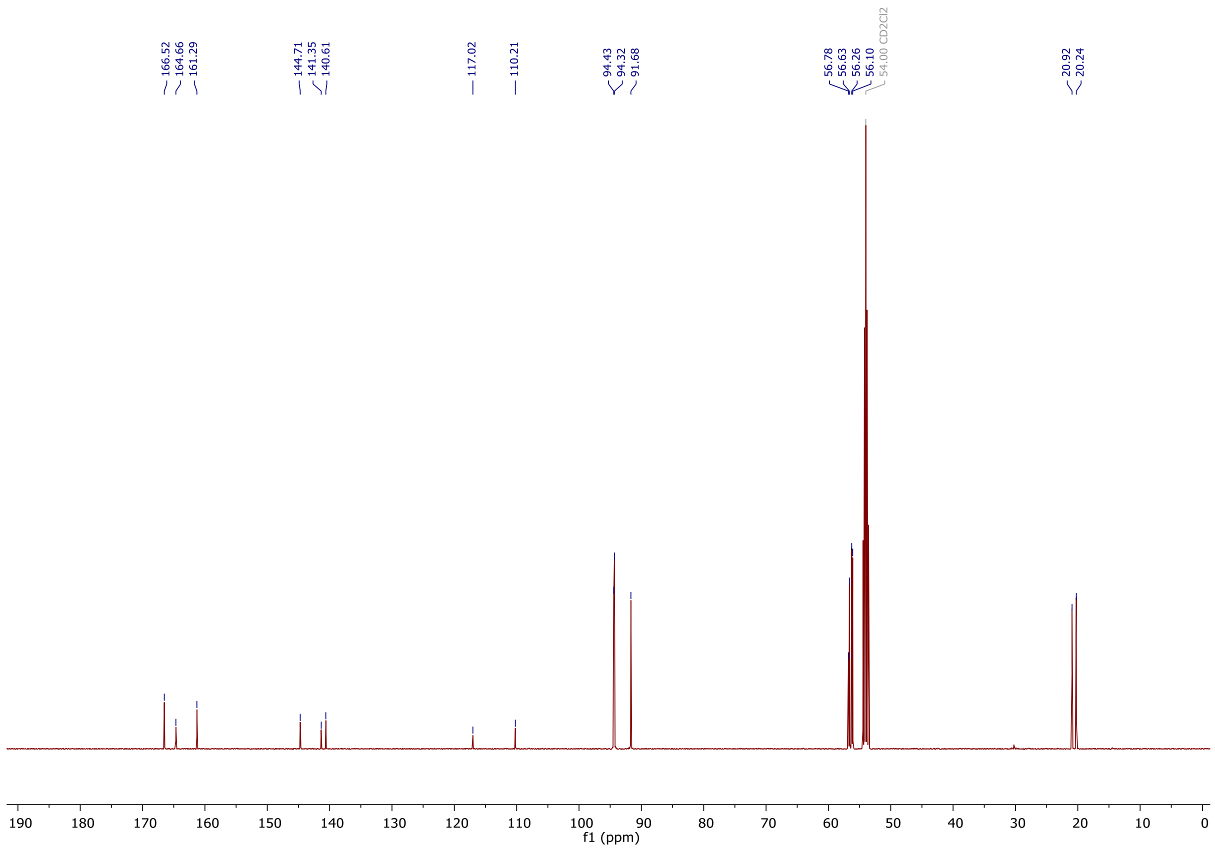

Figure S43. ^{13}C NMR (126 MHz, CD_2Cl_2) spectrum of **2c**.

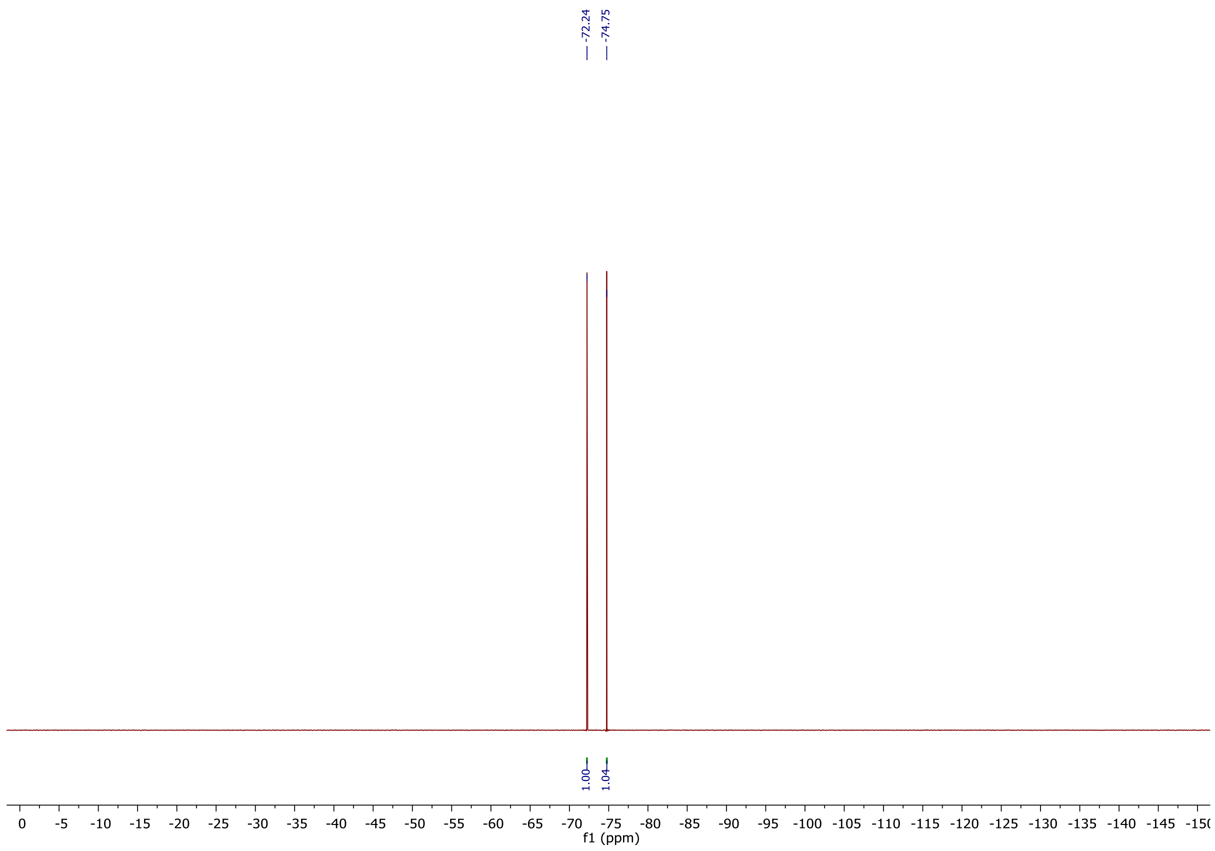

Figure S44. ^{19}F NMR (282 MHz, CD_2Cl_2) spectrum of **2c**.

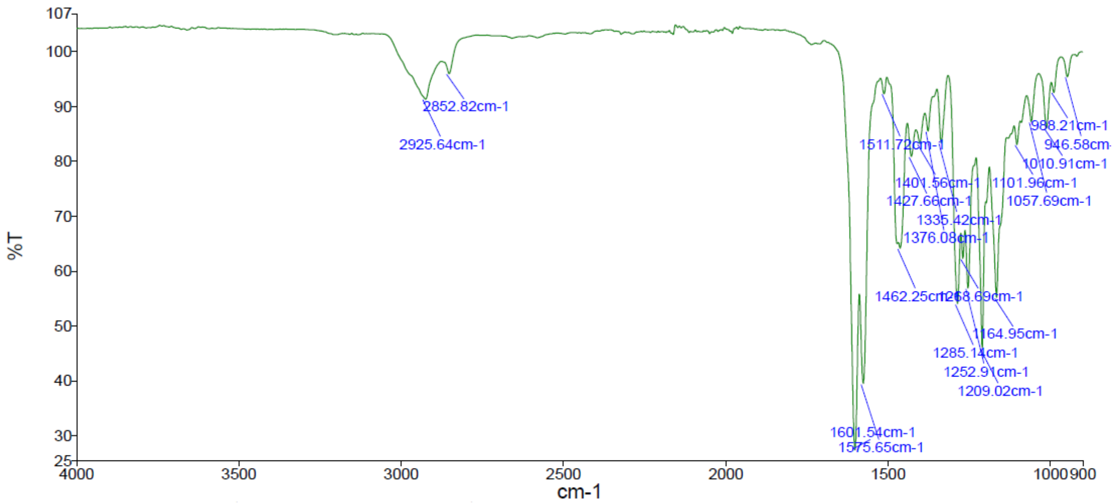

Figure S45. IR (neat) spectrum of **2c**.

Mass Spectrometry Core Facility

Lacour Group – University of Geneva

ESI-HRMS – Certificate of Analysis

Applicant:	Bibiana Fabri	Date of certificate:	03/10/23
Sample name:	BF-NiPr	Instrument:	Xevo G2 Tof (TOF)
Folder:	031023.PRO	Mobile phase:	MeOH (100 µl/min)
Analyst:	Stéphane Grass	Ionisation mode:	ESI (positive polarity)

Elemental Formula	Ion type	Masslynx values *** calc. m/z	Masslynx values *** meas. m/z	Calc. *m/z*	Meas. *m/z*	Accuracy [a] (ppm)
$C_{30}H_{35}N_2O_5$	[M+]	503.2546	503.2542	503.2541	503.2537	-0.8

[a] *Mass spectrum is calibrated by the use of the MS lockspray system (LeuEnk calibration solution).*

**** MassLynx software does not take into account the mass of the electron for ionic species, therefore the shift of m/z 0.000459.*

Zoomed mass spectrum – Isotopic distribution.

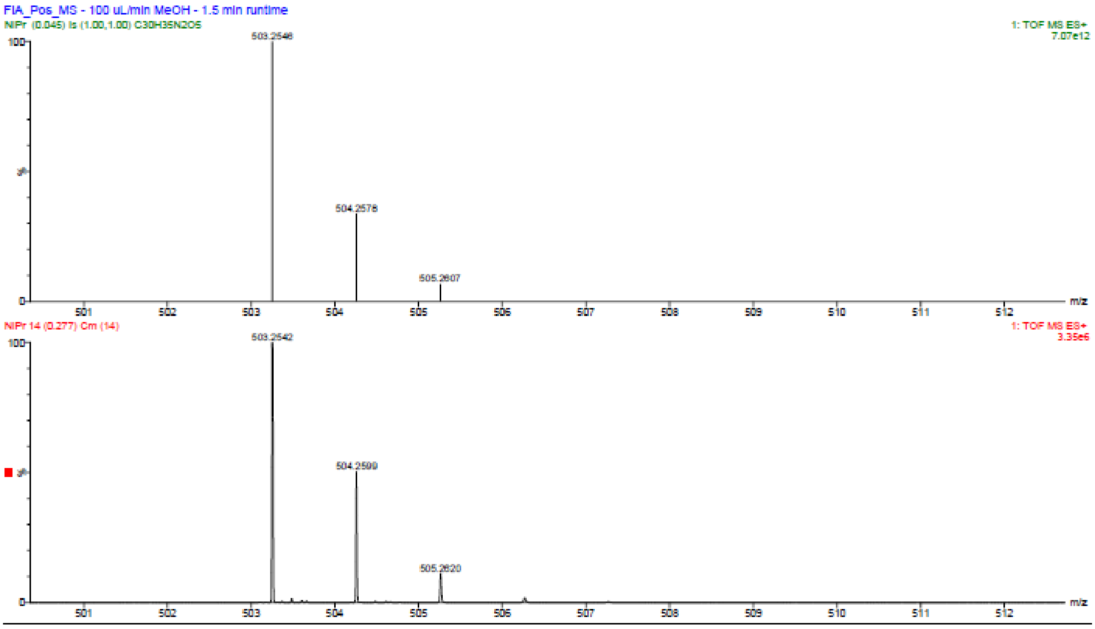

Figure S46. HRMS analysis (ESI, CH_3OH) report of **2c**.

Compound **2d**

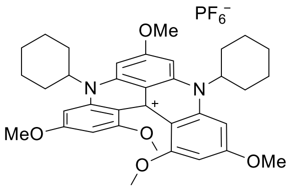

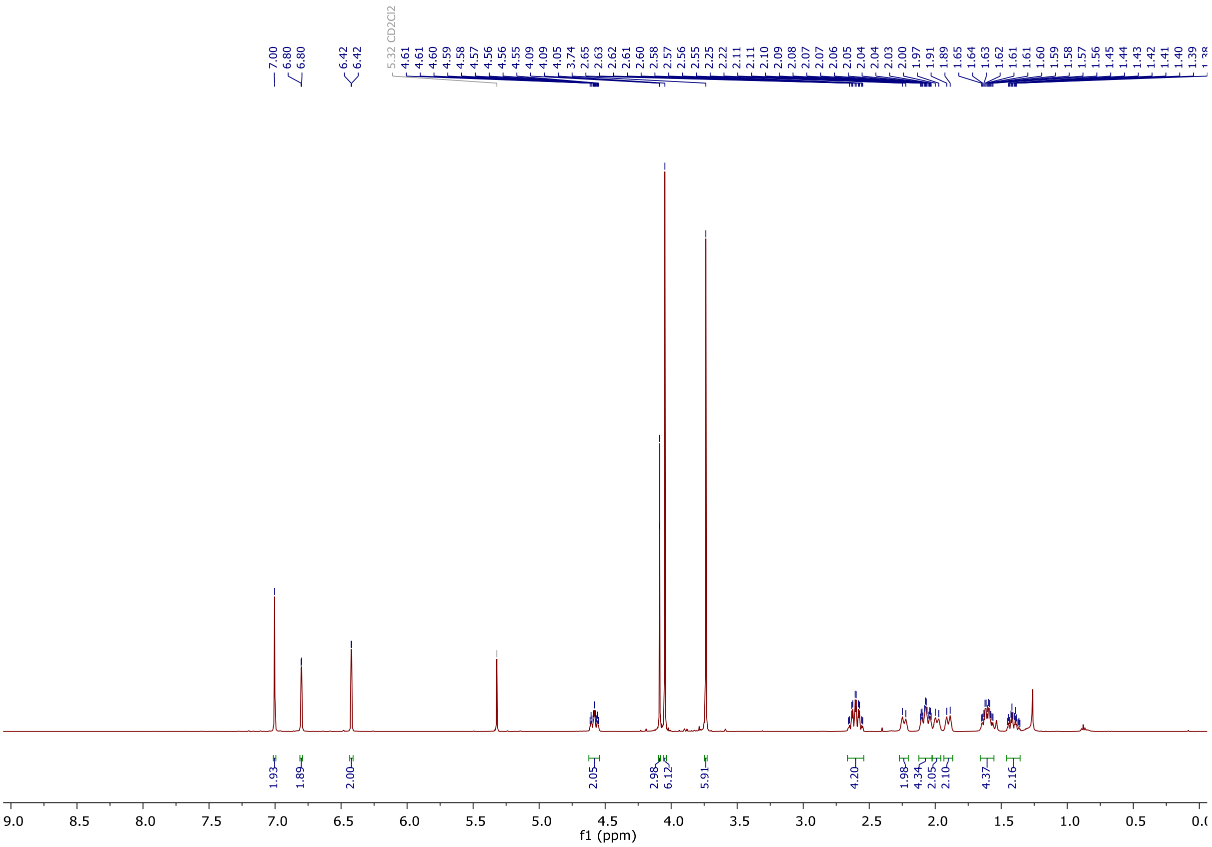

Figure S47. ^1H NMR (500 MHz, CD_2Cl_2) spectrum of **2d**.

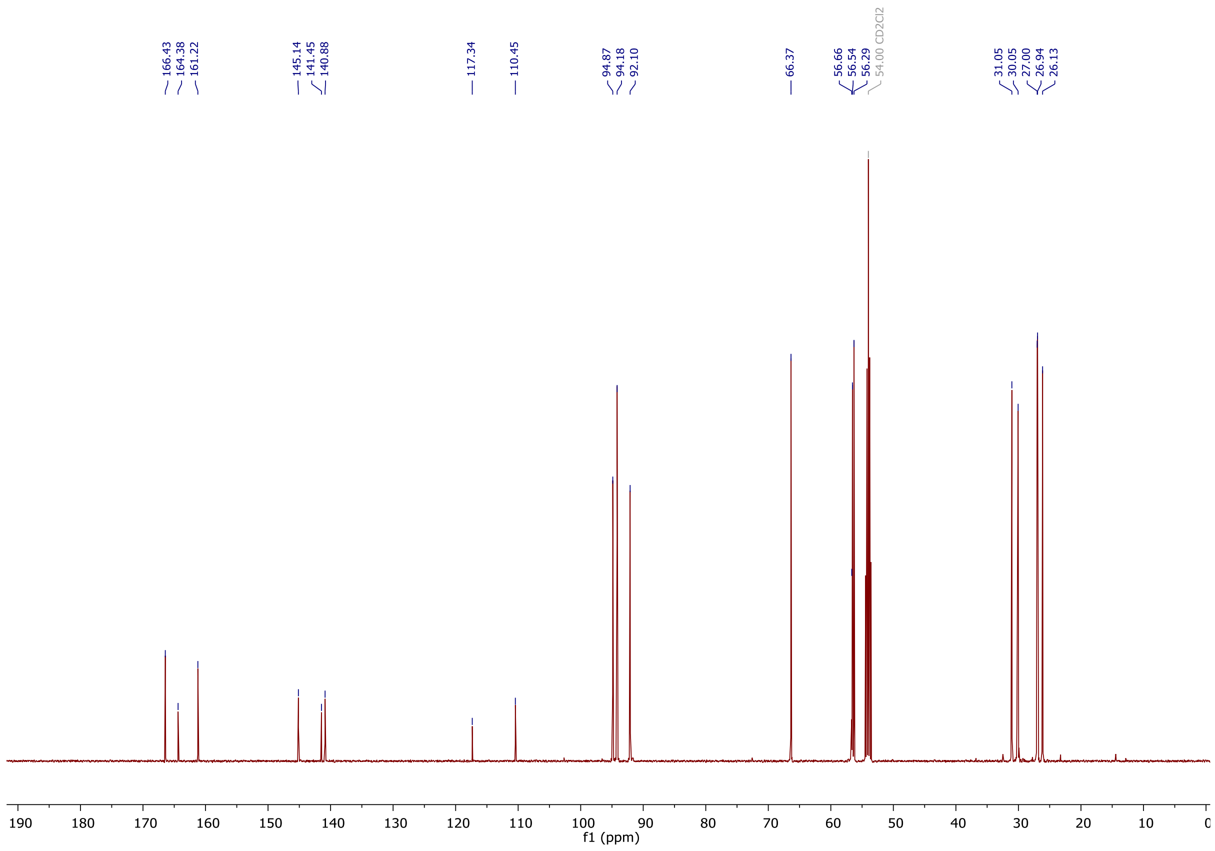

Figure S48. ^{13}C NMR (126 MHz, CD_2Cl_2) spectrum of **2d**.

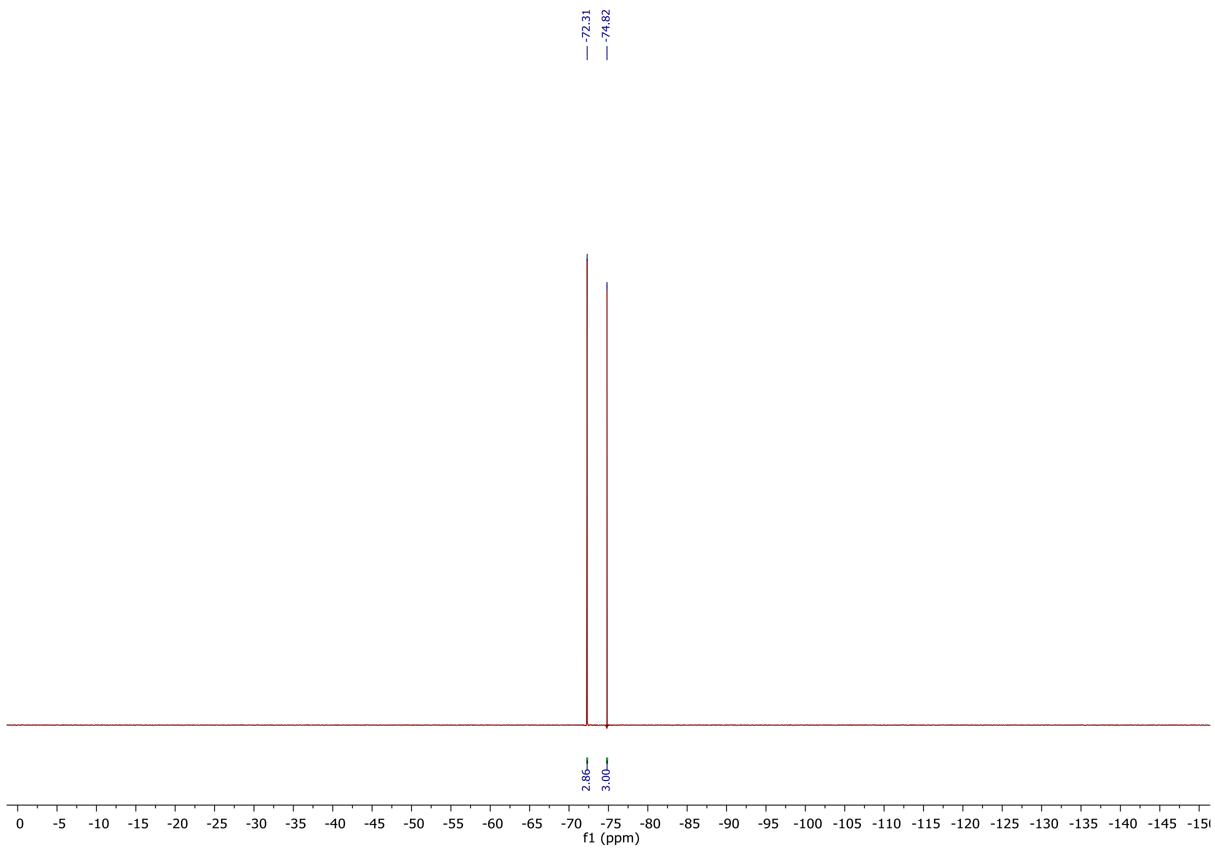

Figure S49. ^{19}F NMR (282 MHz, CD_2Cl_2) spectrum of **2d**.

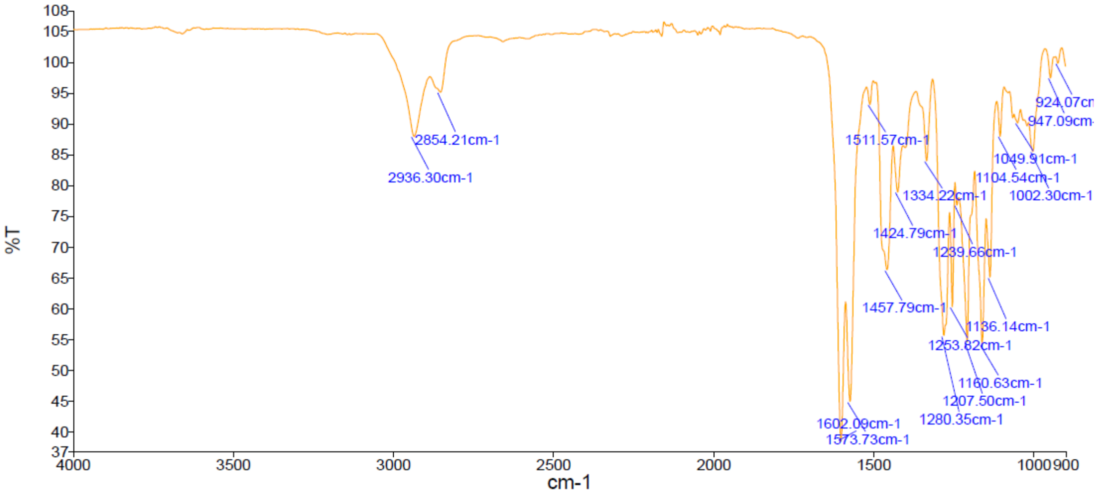

Figure S50. IR (neat) spectrum of **2d**.

Mass Spectrometry Core Facility

Lacour Group – University of Geneva

ESI-HRMS – Certificate of Analysis

Applicant:	Bibiana Fabri	Date of certificate:	03/10/23
Sample name:	BF-NCyHex	Instrument:	Xevo G2 Tof (TOF)
Folder:	031023.PRO	Mobile phase:	MeOH (100 µl/min)
Analyst:	Stéphane Grass	Ionisation mode:	ESI (positive polarity)

Elemental Formula	Ion type	Masslynx values *** calc. *m/z*	Masslynx values *** meas. *m/z*	Calc. *m/z*	Meas. *m/z*	Accuracy [a] *(ppm)*
$C_{36}H_{43}N_2O_5$	[M+]	583.3172	583.3155	583.3167	583.3150	-2.9

[a] *Mass spectrum is calibrated by the use of the MS lockspray system (LeuEnk calibration solution).*

**** MassLynx software does not take into account the mass of the electron for ionic species, therefore the shift of m/z 0.000459.*

Zoomed mass spectrum – Isotopic distribution.

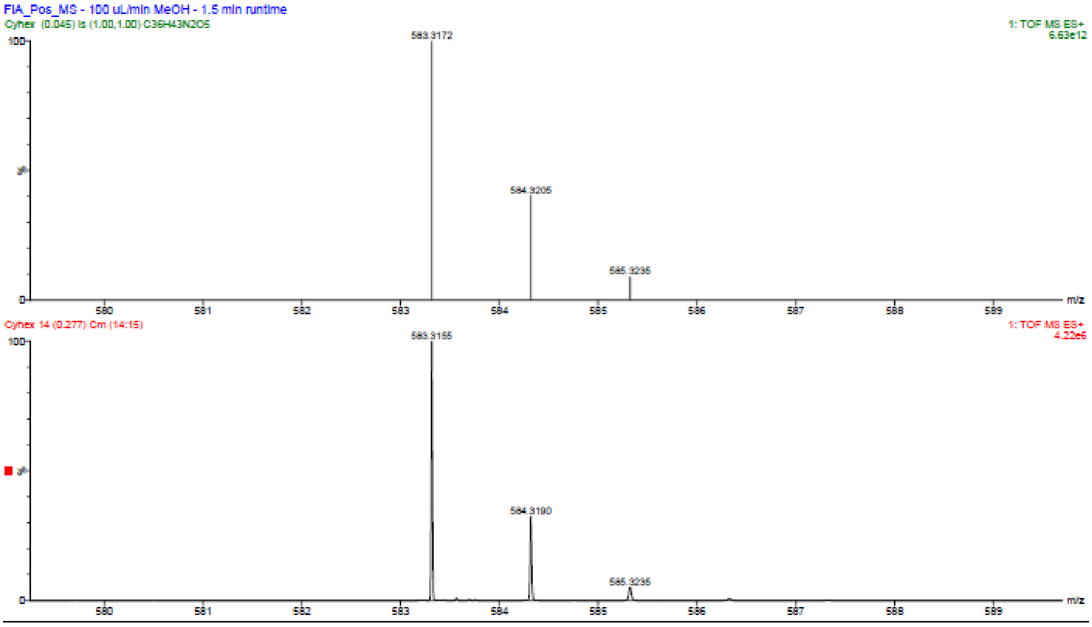

Figure S51. HRMS analysis (ESI, CH_3OH) report of **2d**.

References

1 R. Carr, R. Puckrin, B. K. McMahon, R. Pal, D. Parker and L.-O. Pålsson, *Methods and Applications in Fluorescence*, 2014, **2**, 024007.

2 C. Xu and W. W. Webb, *Journal of the Optical Society of America B*, 1996, **13**, 481-491.

3 L.-O. Pålsson, R. Pal, B. S. Murray, D. Parker and A. Beeby, *Dalton Trans.*, 2007, 5726-5734.

4 L. Young, J. Sung, G. Stacey and J. R. Masters, *Nat. Protoc.*, 2010, **5**, 929-934.

5 R. Pal, *Faraday Discuss.*, 2015, **177**, 507-515.

6 C. A. Schneider, W. S. Rasband and K. W. Eliceiri, *Nat. Methods*, 2012, **9**, 671-675.

7 S. Bolte and F. P. Cordelières, *J. Microsc.*, 2006, **224**, 213-232.

8 (a) C. Herse, D. Bas, F. C. Krebs, T. Bürgi, J. Weber, T. Wesolowski, B. W. Laursen and J. Lacour, *Angew. Chem. Int. Ed.*, 2003, **42**, 3162-3166; (b) N. Mehanna, S. Grass and J. Lacour, *Chirality*, 2012, **24**, 928-935; (c) S. G. Stenspil, A. H. Olsson, R. Mucci, M. Pink, C. Besnard, G. Pescitelli, J. Lacour, A. H. Flood and B. W. Laursen, *Angew. Chem. Int. Ed.*, 2024, e202412320.

9 B. Fabri, T. Funaioli, L. Frédéric, C. Elsner, E. Bordignon, F. Zinna, L. Di Bari, G. Pescitelli and J. Lacour, *J. Am. Chem. Soc.*, 2024, **146**, 8308-8319.
